# How newspaper images position different groups of people in relation to the COVID‐19 pandemic: A social representations approach

**DOI:** 10.1002/casp.2515

**Published:** 2021-03-04

**Authors:** Jari Martikainen, Inari Sakki

**Affiliations:** ^1^ Department of Social Sciences (Social Psychology) University of Eastern Finland Kuopio Finland

**Keywords:** age groups, media images, positioning, social representations, visual rhetoric

## Abstract

This study examines newspaper photographs related to the COVID‐19 pandemic in Finland. Drawing on social representations theory and positioning theory, we explore social representations and identities related to COVID‐19 in mass media using a visual rhetoric analysis. More specifically, we focus on how newspaper photographs construct subjects' positions for different age groups. The data consisted of 4,506 photographs of people published in the two largest Finnish newspapers between 1 January and 31 August 2020. The study identified the following subject positions for the different age groups: (a) children as controlled pupils and joyful players; (b) youth as future‐oriented graduates and reckless partygoers; (c) adults as authoritative experts, adaptive professionals, responsible caretakers and active recreationists and (d) elderly people as isolated loners. In addition to echoing the positions of villains, heroes and victims identified in previous studies, the photographs seemed to construct an intergroup divide between adults and the other age groups. Methodologically, this study elaborates the usefulness of the analysis of visual rhetoric in social representations research. Theoretically, we seek to advance the understanding of the role of media, particularly images, in the social construction of knowledge.

## 1 INTRODUCTION

Since the outbreak of the COVID‐19 pandemic, the disease has gained massive media attention throughout the world. The origin, causes and nature of the virus; its symptoms and fatality; treatment; safety measures to protect from infection; vaccination development and the pandemic's effect on national and international economies and people's wellbeing are some of the topics that have gained vivid media exposure. This is also the case in the Finnish media, which is the context of the present study. There have been numerous international studies on exposure to COVID‐19 news through social media and its influence on people (see Allington, Duffy, Wessely, Dhavan, & Rubin, 2020; Nabity‐Grover, Cheung, & Thatcher, 2020; Pennycook, McPhetres, Zhang, Lu, & Rand, 2020), but to our knowledge, no studies have yet focussed on COVID‐19‐related newspaper images, which is the focus of this study.

Despite the number of people who have been infected by coronavirus, the majority of the population has not been infected and must therefore form their conceptions of the disease based on news, media and anecdotes. It is this combination of own and other people's experiences, news and media stories and images that provide people with the ingredients to construct their understanding of the COVID‐19 pandemic. Among these ingredients, news stories are often regarded as objective information and news images as their visual proof, and for this reason, their impact is considerable (Banks, 2012; Zelizer, 1995).

Social representation theory (SRT) provides the theoretical framework for studying communities' construction of social knowledge (Howarth, 2001; Moscovici, 1961/2008). Thus far, few studies have used the SRT approach for the study of COVID‐19 (see Apostolidis, Santos, & Kalampalikis, 2020; De Rosa & Mannarini, 2020; Idoiaga, Berasategi, Eiguren, & Picaza, 2020; Jaspal & Nerlich, 2020; Kouame, Digbeu, & Samouth, 2020; Páez & Pérez, 2020; Pizarro, Michael, & Mhaskar, 2020). Many of these studies (e.g., Apostolidis et al., 2020; Jaspal & Nerlich, 2020; Páez & Pérez, 2020) were short theoretical papers, and empirical studies of COVID‐19 using SRT are still scarce, although this is likely to change in the near future as more studies get published (Colì, Norcia, & Bruzzone, 2020; Emiliani et al., 2020; Pizarro et al., 2020). Our study aims to contribute to this emerging discussion on social representations of COVID‐19. We based our examination on a large sample of newspaper photographs published in the two largest Finnish newspapers during the first 8 months of the pandemic. Our aim is to investigate the ways in which representations and identities are constructed in newspaper photographs portraying the COVID‐19 pandemic. To do so, we draw from positioning theory (Davies & Harré, 1990; Harré & Moghaddam, 2003) to determine the subject positions constructed for different groups of people in relation to social representations of COVID‐19. How social representations and identities are built and used is foundational to interpersonal and intergroup relations, solidarity versus antagonism and social stability vs. community conflict.

Empirically, our study provides new knowledge about how different groups of people are represented and positioned vis‐à‐vis COVID‐19. Methodologically, by building upon the visual rhetorical approach, we elaborate the usefulness of analysing visual rhetoric in social representations research. Theoretically, our study seeks to advance the understanding of the role of media, particularly images, in the social construction of knowledge.

### 1.1 Social representations of the pandemic and positioning

Based on community psychology and social knowledge, SRT provides a theoretical framework to study the transmission of knowledge from scientific thinking to lay thinking within communities via mass media. According to Moscovici (1961/2008), crises and upheavals stir the order of societies and are thus exceptionally fruitful for the study of social representations. Previous research has shown the usefulness of SRT in the study of emerging epidemics, such as SARS (Washer, 2004), mad cow disease (Washer, 2006), Ebola (Joffe & Haarhoff, 2002), HIV/AIDS (Jaspal & Nerlich, 2016; Joffe & Bettega, 2003), Zika (Ribeiro, Hartley, Nerlich, & Jaspal, 2018) and the H1N1 epidemic (Wagner‐Egger et al., 2011). A common finding in these studies was that the social construction of knowledge is intertwined with the process of othering. In these studies, the position of the other was usually assigned to groups of people, often foreigners or out‐groups within a society, who were among the first infected by the epidemic. These others were blamed for the pandemic, believed to be at risk because of their dirty practices or immoral behaviour or accused of intentionally spreading the disease (Joffe, 1999; Washer, 2006). Joffe (2011) stated that the public response to emerging infectious diseases follows the distancing‐blame‐stigma pattern, in which the disease is first distanced from the one's in‐groups, after which particular people or groups are blamed for the disease's origin and spread and finally those who have contracted it are stigmatised.

SRT holds that making sense of new ideas in society is carried out in three processes: new ideas are anchored within established cultural understandings; objectified with tangible symbols, images or metaphors and naturalised when they are repeated in social interactions and practices, to the extent that they become taken for granted (Hakoköngäs & Sakki, 2016; Moscovici, 1984). Páez and Pérez (2020) described how the anchoring of the representations of COVID‐19 has taken place in relation to past diseases (Spanish flu and SARS), other nationalities (Chinese) and anti‐hygienic practices. In contrast, Jaspal and Nerlich (2020) suggested that COVID‐19 has been anchored to HIV in media reporting and objectified in war metaphors such as combat, fight and defeat, which can then be used in the justification of harsher restrictions and fighting the enemy at all costs.

The role of media communication in the dissemination of ideas and construction of lay understanding has been at the centre of SRT since the seminal work by Moscovici (1961/2008). By informing about, reporting on and discussing social phenomena in words and images, media stories and images simultaneously materialise social representations in verbal and visual forms; shape people's thoughts, actions and emotions (Moscovici, 1984) and position people in relation to the phenomena in question (Phoenix, Howarth, & Philogène, 2017). The power of mass media occurs not only through the construction and dissemination of social representations but also through meta‐representations, that is, what we believe that significant others (ingroup or outgroup members) think (Elcheroth, Doise, & Reicher, 2011). When people read the same newspaper and view the same images, they may remain doubtful about what they see but still be influenced by the impact they believe that media has on other people (Elcheroth et al., 2011).

Social representations – as systems of values, ideas and practices – establish social order and participate in the construction of social identities (Howarth, 2002; Phoenix et al., 2017). This positioning potential of social representations is the focus of this study. Reminiscent of Harré and Moghaddam's (2003) positioning theory, Phoenix et al. (2017) argued that social representations position people in relation to each other. Social representations are not ‘quiet things’ (Howarth, 2006; Moscovici & Marková, 1998) that are disinterested or neutral but rather are constructed from numerous motives and positions; thus, social representations construct power relations between people and groups. Andreouli (2010) integrated SRT and positioning theory to suggest that the concept of positioning is the essential link between social representation and identity. By drawing on positioning theory, it follows that every subject position has a moral component and is associated with a set of rights and duties, and these identity positions create power dynamics (Davies & Harré, 1990; Harré & Moghaddam, 2003).

This basic idea of positioning theory echoes previous studies related to social representations of outbreaks, which found that the process of representation and identity construction involves a moral dimension and power dynamics. Different group of peoples, or collectives, fulfil important symbolic functions in coping with suddenly emerging threats like disease outbreaks. In relation to representations of COVID‐19, different groups are positioned differently as heroes, villains and victims (Wagner‐Egger et al., 2011). Páez and Pérez (2020) suggested that the position of heroes of COVID‐19 is assigned to healthcare workers, villains to careless people and victims to the elderly and poor. This sensemaking and positioning is action; it creates solidarity and antagonism between social groups in communities. Due to their visual concreteness and capability of appealing to emotions, images are powerful means of communication (Blair, 2004). Therefore, it is of utmost importance to examine the ways in which media images of COVID‐19 construct roles and identities (i.e., subject positions) for different groups of people. Drawing from the analysis of visual rhetoric, this paper explores the visual strategies employed in newspaper images to construct subject positions for different groups of people.

Recent research on social representations has increasingly focussed on the role of visual materials and communication in the process of social representation (Hakoköngäs, 2017; Martikainen, 2020; Sakki, 2010). Already Moscovici (1961/2008) acknowledged the importance of visuals in his seminal work on SRT, and De Rosa (1998, 2001) continued this line of research by addressing iconic and visual modes of persuasion in the investigation of social representations of the Benetton brand. De Rosa (2014) later introduced her modelling approach to social representations. According to De Rosa and Farr (2001), the usefulness of visual materials in social representations research is based on the fact that visuals can activate (anchoring), express (objectification) and disseminate (naturalisation) social representation. For the purposes of the present study, processes of objectification and naturalisation are the most salient. The visual concreteness of images and their ability to evoke emotions make them powerful means of communication and persuasion (Blair, 2004; Moliner, 2020).

## 2 METHOD

### 2.1 Data

The data in this study are photographs published in two Finnish newspapers with nationwide circulation, *Helsingin Sanomat* and *Ilta‐Sanomat*. *Helsingin Sanomat* is the largest circulated newspaper in the Nordic countries, with a total daily distribution of 339,437 copies in 2019 (Media Audit Finland, 2019). *Ilta‐Sanomat*, in turn, is the largest tabloid newspaper, with 2.65 million readers each week throughout the Finnish nation (https://www.is.fi/kotimaa/art-2000006442856.html). Both newspapers are published in print and digital formats. Because of their large circulation and nationwide readership, it can be concluded that the news and images published in these outlets influence the way people perceive and understand the COVID‐19 pandemic and related topics.

A total of 9,558 visual images related to the COVID‐19 pandemic were published in the two newspaper between 1 January and 31 August 2020. Of these, 7,857 images dealt with the pandemic in Finland. Since we focussed on photographs of people, we ruled out other subject matter and other types of images (e.g., charts, drawings, graphic works and advertisements). Thus, the data consisted of 4,508 photographs of people in relation to the COVID‐19 pandemic in Finland.

### 2.2 Analytic procedure

In this study, we focussed on those COVID‐19‐related newspaper images that portrayed people. After preliminary bottom‐up reading of our data, we decided to organise the groups of people according to broad age groups. It should be noted that prior to this analysis, we had planned to organise our data differently, for example, we were curious about the ways minority groups would be visualised in newspaper images. However, there were relatively few images related to minorities or out‐groups (which is an interesting result in itself). We instead found a strong intergenerational pattern throughout the visual narration, and thus we decided to organise our data according to (loose) age groups, which would then allow us to delve deeper into the visual rhetorical analysis of different subject positions constructed for the different generations.

The analysis of visual rhetoric focusses on images' contents, form and functions (Danesi, 2017; Foss, 2005), and for this reason, it is congruent with the field of social representations research (Hakoköngäs & Sakki, 2016; Martikainen, 2020). Regarding the form of the image, the analysis of visual rhetoric not only pays attention to people, objects and environments but also to the visual means of expression – such as form, colour, composition, viewing angle, light and shadow, proximity and framing – that create a stance regarding the people, objects and environments (Foss, 2005; Martikainen, 2019). In our study, we focussed on exploring these means of visual expression as visual rhetorical strategies that position people in different age groups in particular ways.

The analysis of visual rhetoric combines content analysis and (social) semiotic analysis (Martikainen, 2019). First, the visually perceivable elements in the image are analysed by content analysis, paying attention to the subject matter and means of visual expression (Rose, 2016). This is followed by (social) semiotic analysis wherein the findings of the content analysis are interpreted based on a culturally available matrix of meanings (Jewitt & Oyama, 2004; Kress & van Leeuwen, 2006). Even though the separation of the two stages clarifies the procedure, in practice, the two stages are interlaced in the process of analysis. The analysis of visual rhetoric shows that images are not objective replica of reality but created through a number of means of visual expression (Barthes, 1977; Rose, 2016), which can be used to construct certain subject positions for the people depicted. In addition, analysis of visual rhetoric understands that meanings are not in the images themselves but constructed by the perceivers (Kress & van Leeuwen, 2006). Even though there might be culturally preferred ways of interpreting images (Danesi, 2017; Kress & van Leeuwen, 2006), it must be remembered that perceivers' motives, backgrounds and social representations influence the interpretation. This applies to researchers as well, and thus, we do not claim to present objective findings but contextual interpretations based on close reading of images. For this reason, we include a rich set of images in this study to elaborate our reasoning and allow the readers to evaluate our findings. Both authors collaboratively identified subject positions and related visual rhetorical strategies and selected illustrative images for further analysis. The first author was primarily responsible for writing the analysis.

## 3 FINDINGS

The two newspapers published a total of 4,506 photographs of people in relation to COVID‐19 in Finland during the studied time period. We grouped the images of people into four age groups: children (up to 12 years), youth (13 to early 20s), adults (mid 20s to late 60s) and elderly people (from 70 onwards). We admit that it is not always easy – or even possible – to judge people's ages perfectly by their images. Even though captions and news stories helped in many cases to solve uncertainties concerning age, the roughness of the groupings should be kept in mind. Table 1 summarises the number of images depicting people in different age groups.

**TABLE 1:** Distribution of age groups in the images

Age group	Number of images
Single generation	
Only children	193
Only youth	187
Only adults	3,754
Only elderly people	189
Intergenerational	
Children and adults	143
Children and elderly	7
Youth and adults	2
Youth and elderly	4
Elderly and adults	27

The following section presents our findings on the visual rhetorical strategies used to construct particular subject positions for the different age groups. The examples presented are those that offer clear illustrations of the findings. Because of the large number of images – especially for the adult age group – the visual strategies mentioned in each subject position must not be understood as exhaustive but rather as typical examples. Our interpretation of the elements of visual rhetoric was based on the work by Kress and van Leeuwen (2006).

### 3.1 Children: Controlled pupils and joyful players

**FIGURE 1:**
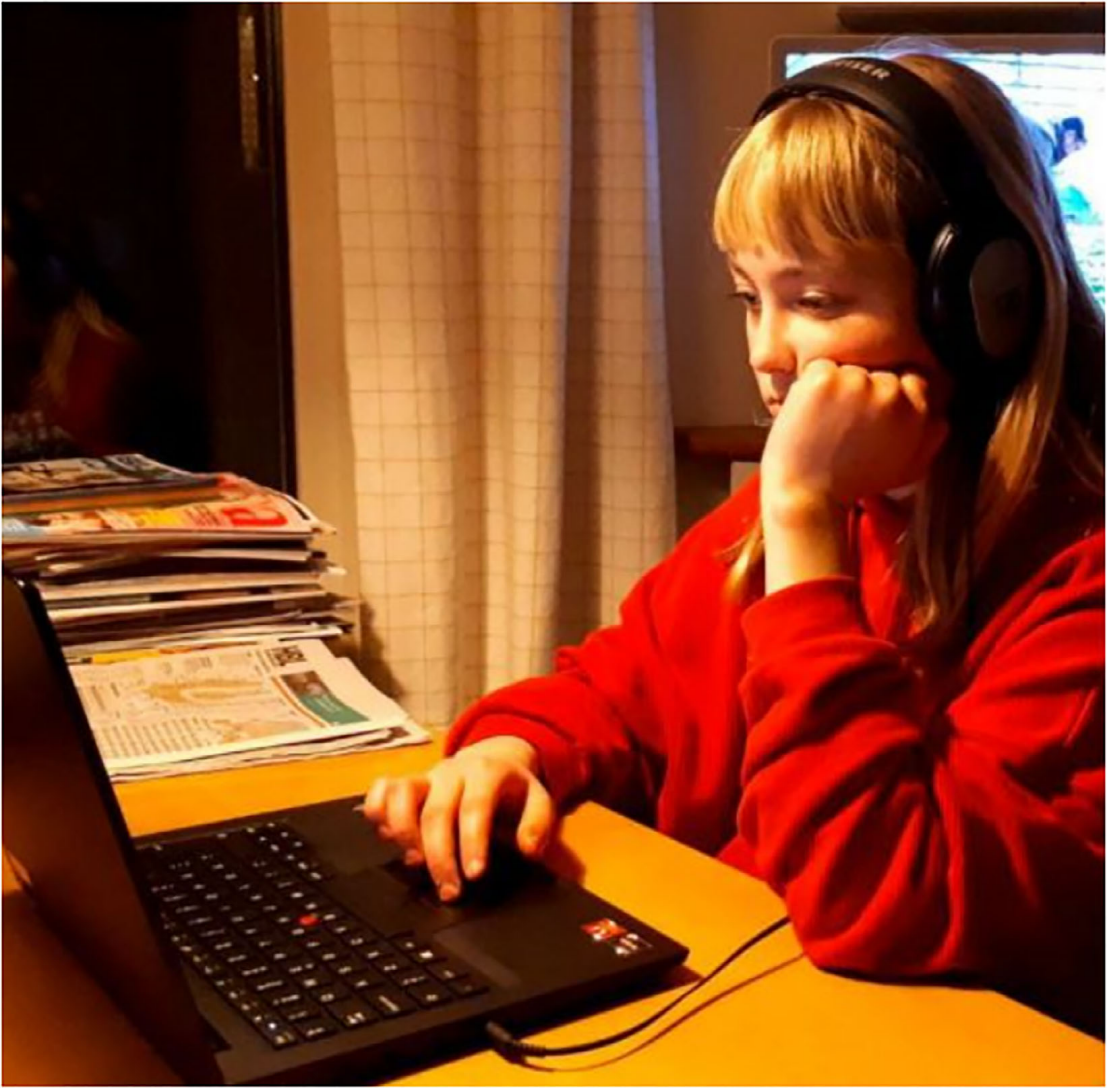
*Helsingin Sanomat* 31.3.2020. Photo: Sini Castren

**FIGURE 2:**
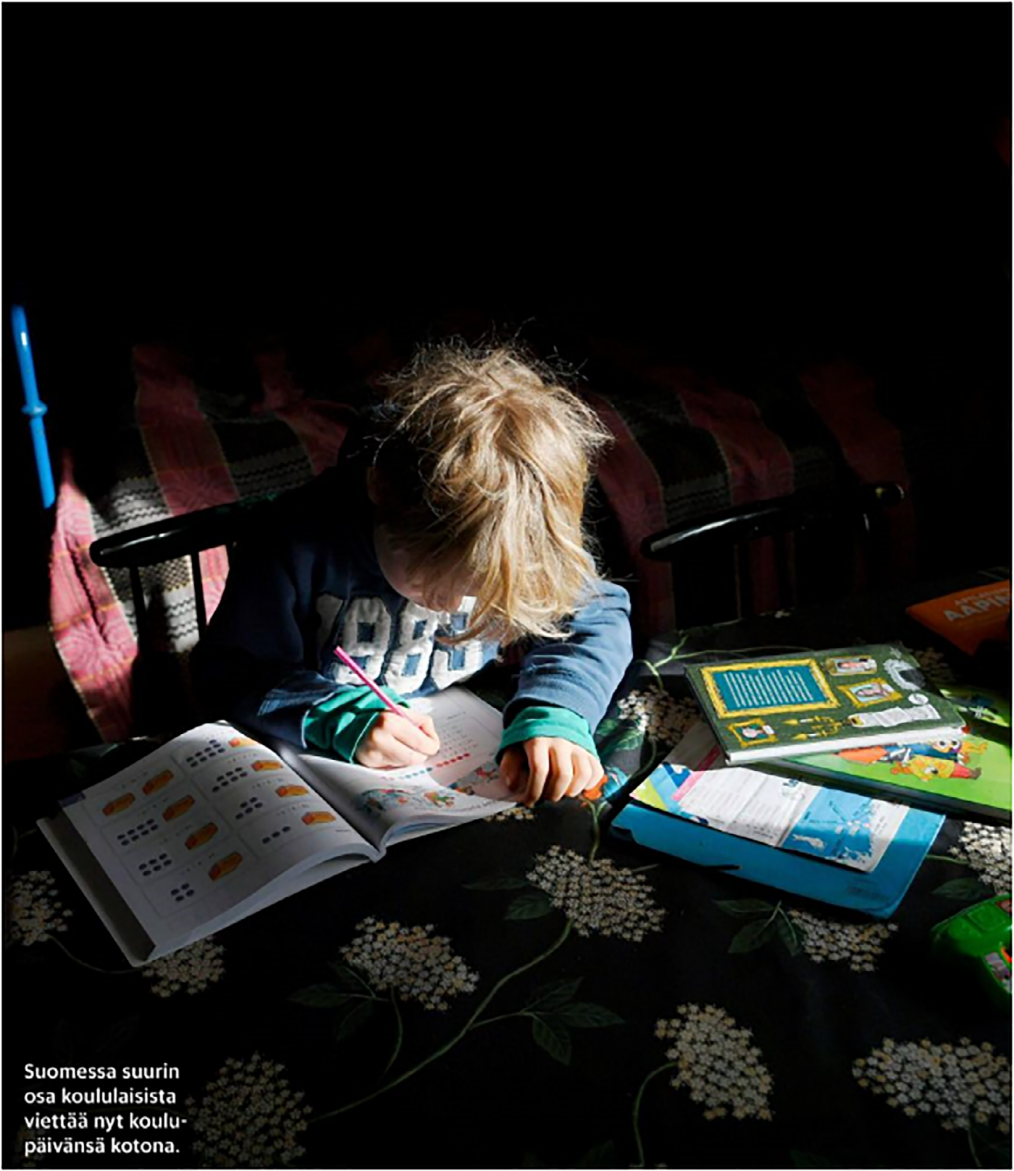
*Helsingin Sanomat* 28.4.2020. Photo: Antti‐Aimo Koivisto/Lehtikuva

**FIGURE 3:**
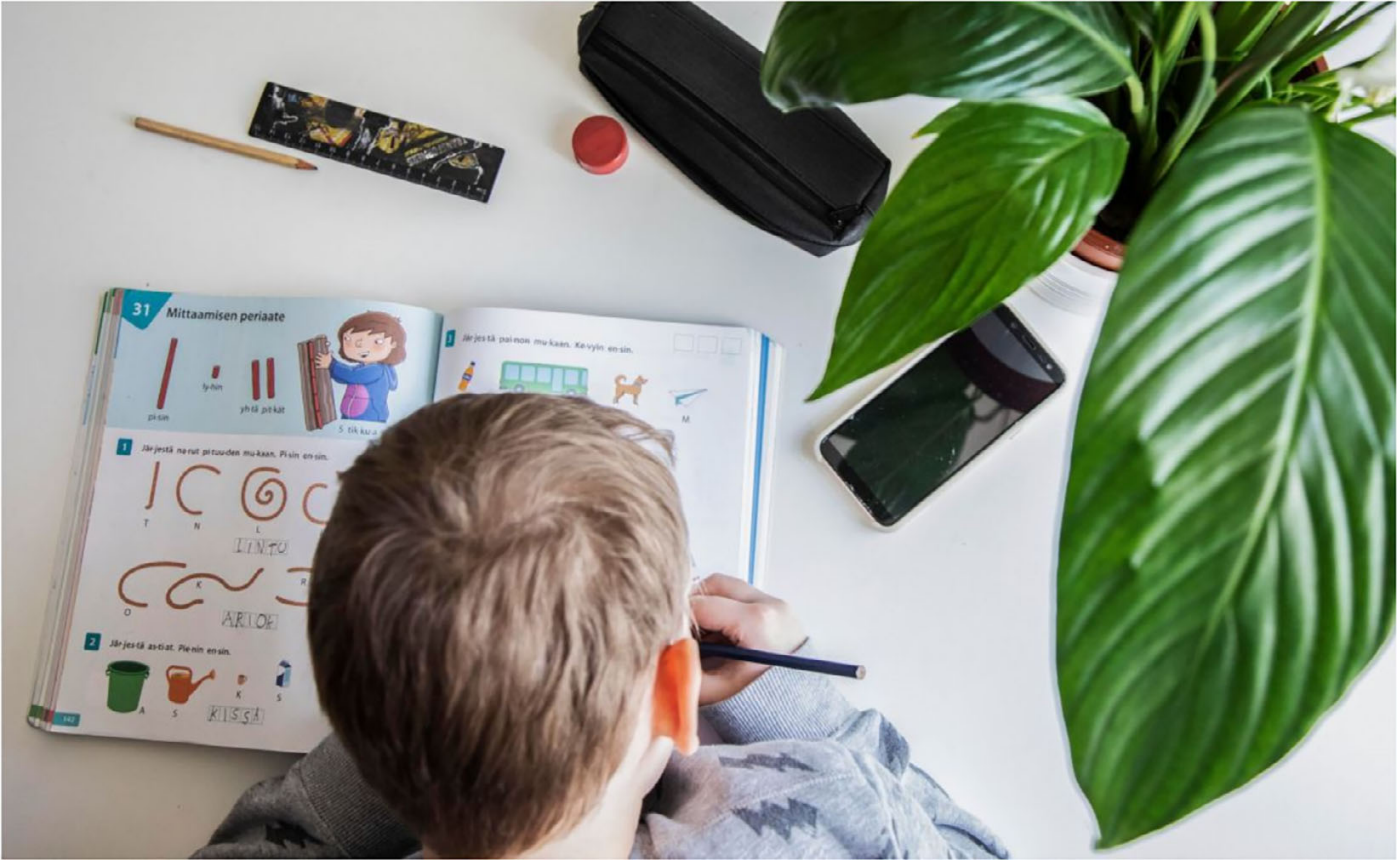
*Helsingin Sanomat* 27.4.2020. Photo: Aleksi Jalava

The majority of the images depicting children are related to learning and school: either distance learning at home or learning at school after the lockdown ended. Children are depicted doing their schools tasks. Images feature objects that emphasise learning aspects, such as computers, books, notebooks, pens, pencils and rulers (Figures 1, 2 and 3). Usually, there is only one child in the image, who has a serious facial expression (Figure 1), which constructs the feeling of loneliness as well as the absence of peers and the social aspect of learning. Figure 2, in which a child is studying in a dark room with only a shaft of light, highlights isolation and loneliness. The darkness may also represent the difficulties of distance learning – a topic frequently discussed in media last spring. In addition to seriousness and isolation, several photos communicate feelings of order and control. They are constructed through the viewing point, namely a high angle, which positions the spectator higher than the child and suggests the spectator is an adult person (Figures 2 and 3). The child appears as inferior, and the spectator (adult) is superior and oversees the child's fulfilment of their school tasks.

**FIGURE 4:**
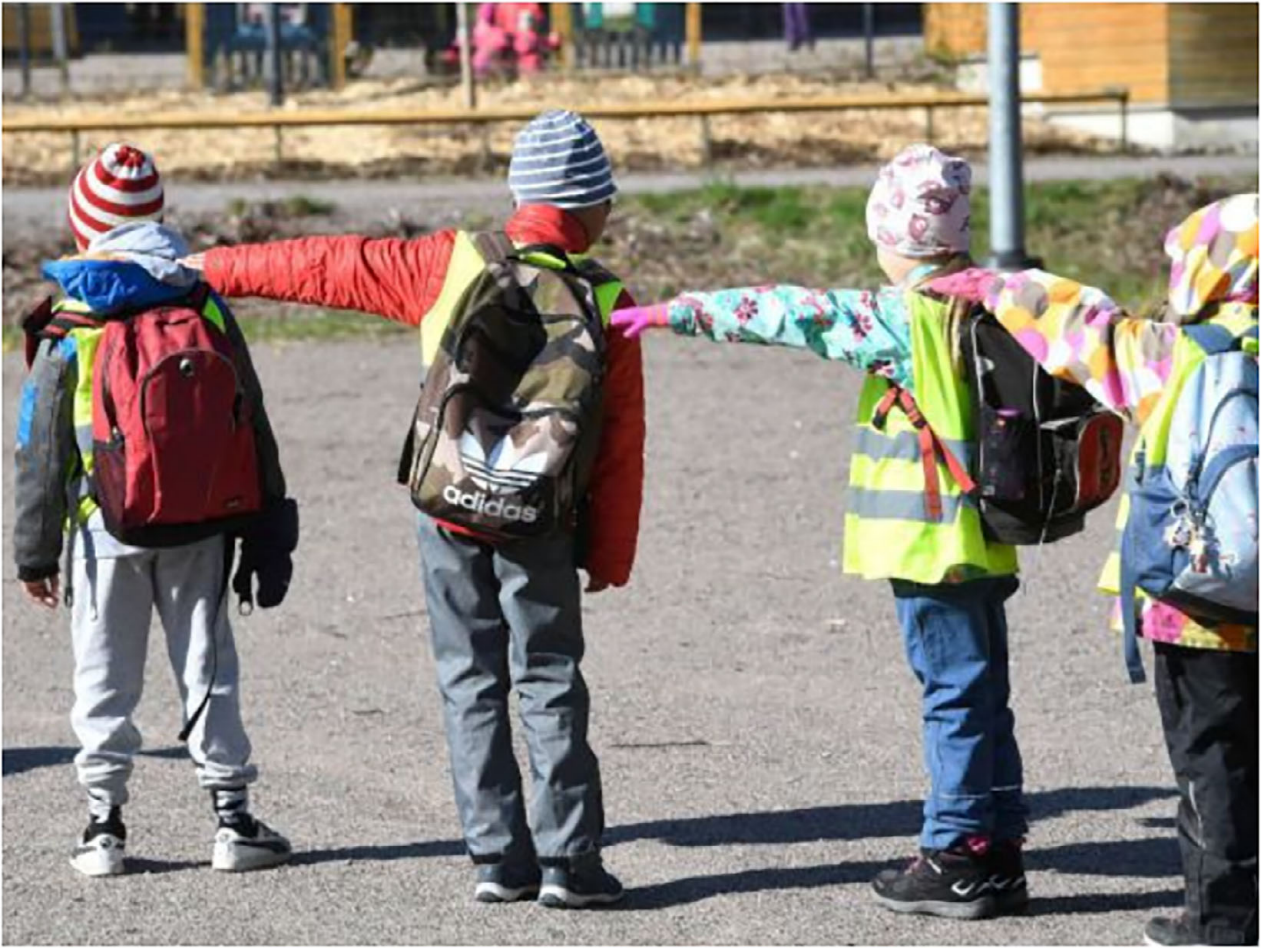
*Helsingin Sanomat* 15.6.2020. Photo: Heikki Saukkomaa/Lehtikuva

**FIGURE 5:**
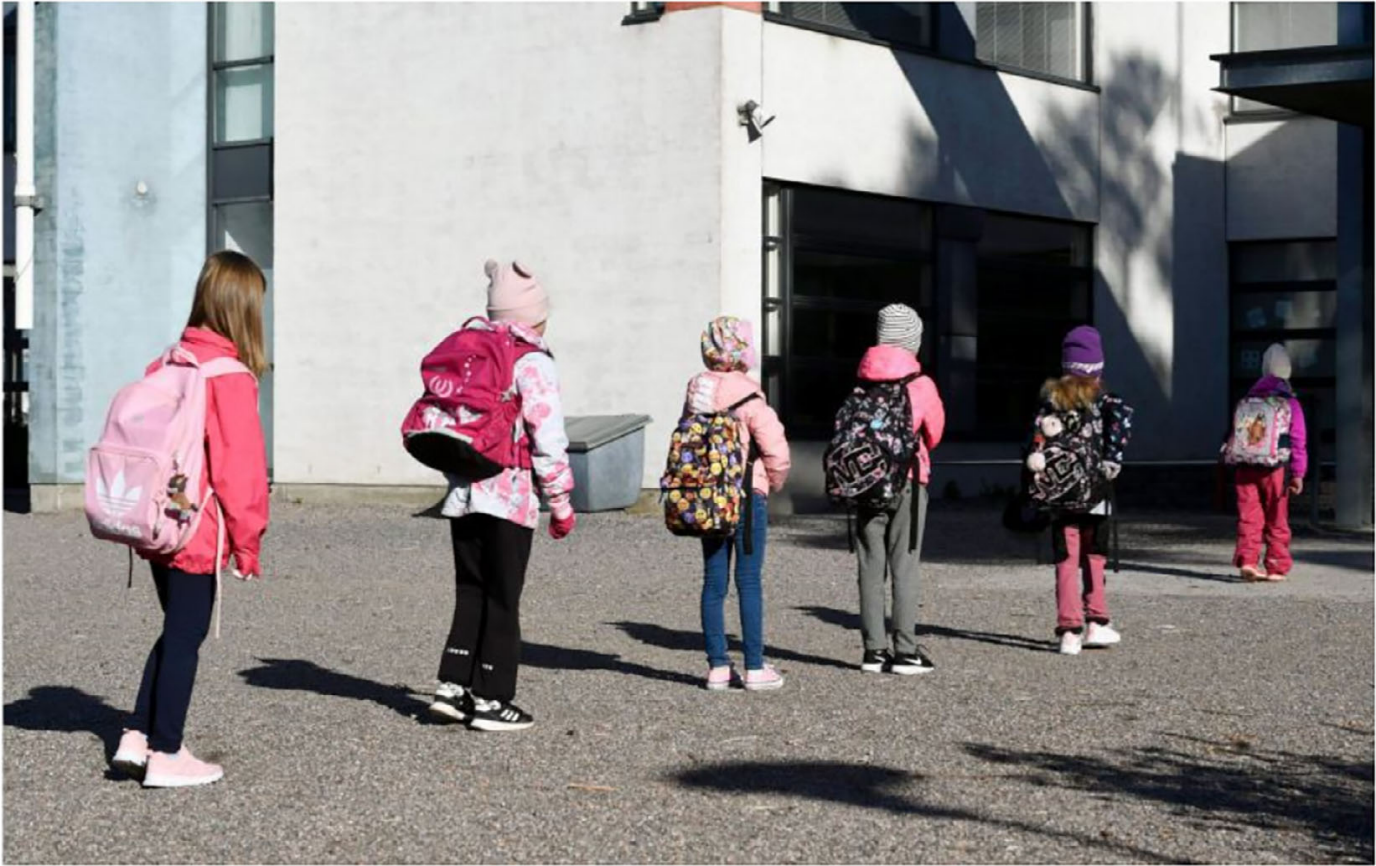
*Helsingin Sanomat* 27.5.2020. Photo: Emmi Korhonen/Lehtikuva

**FIGURE 6:**
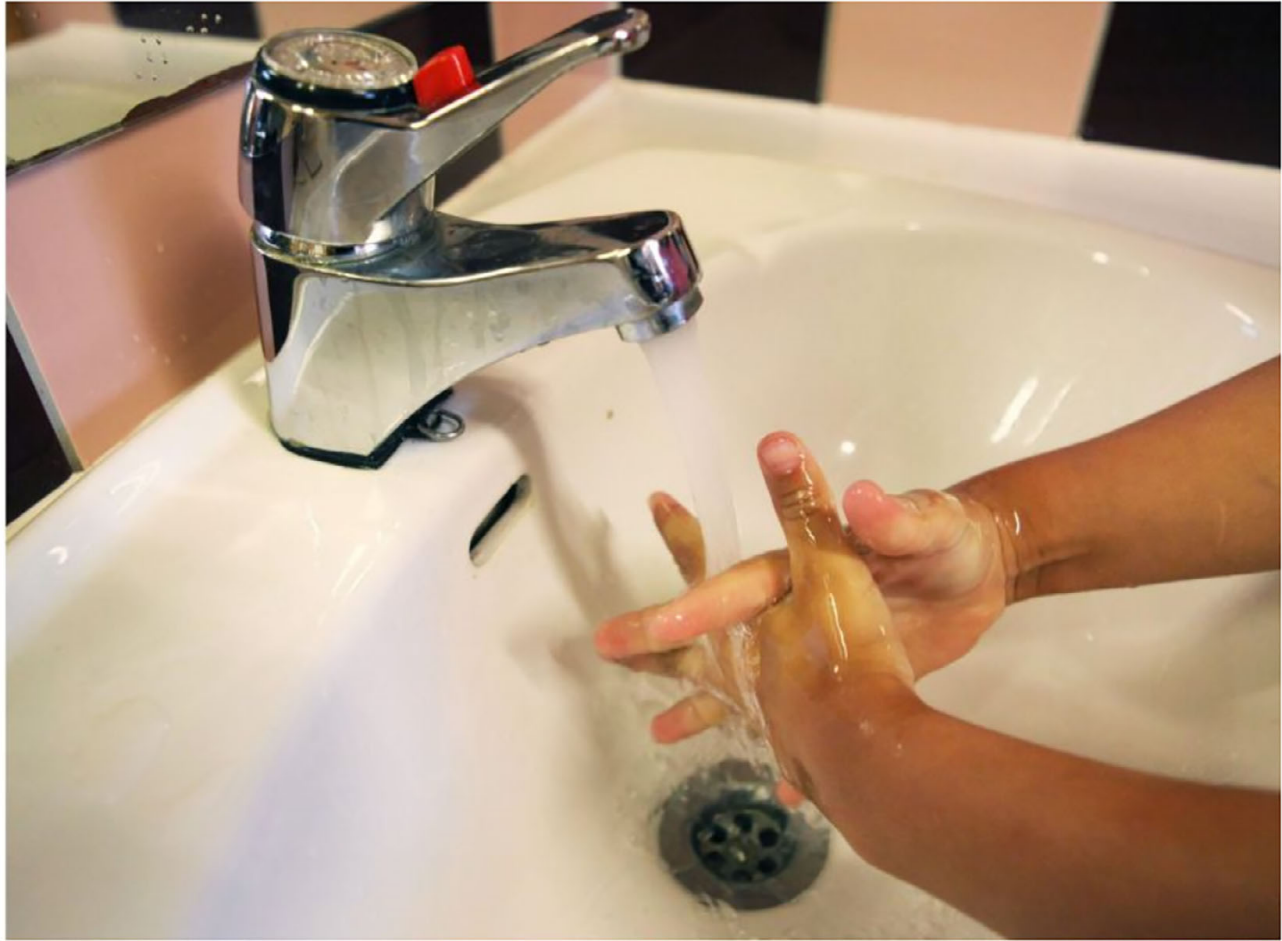
*Helsingin Sanomat* 29.5. 2020. Photo: Juhani Niiranen/HS

**FIGURE 7:**
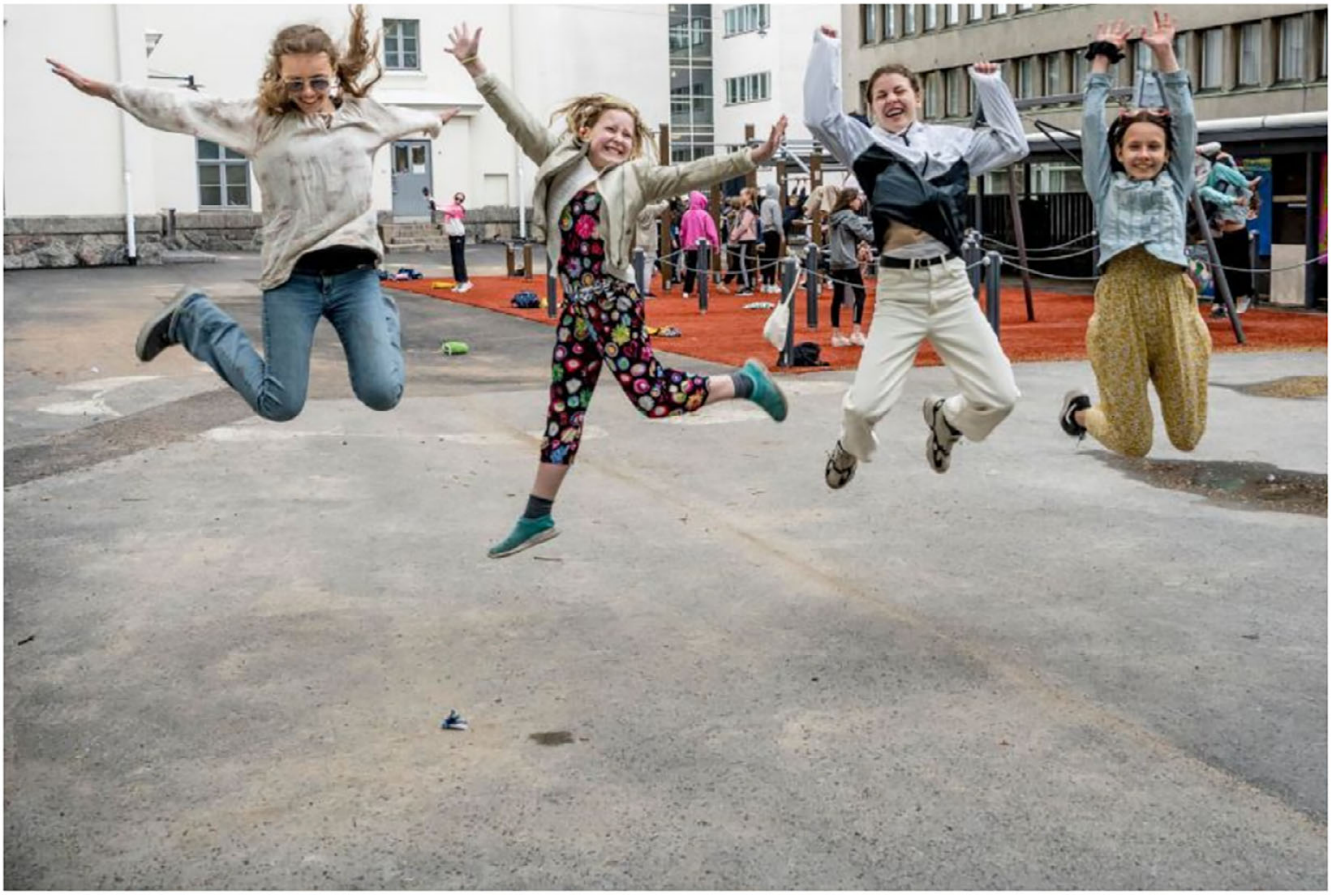
*Helsingin Sanomat* 29.5.2020. Photo: Kari Pullinen

**FIGURE 8:**
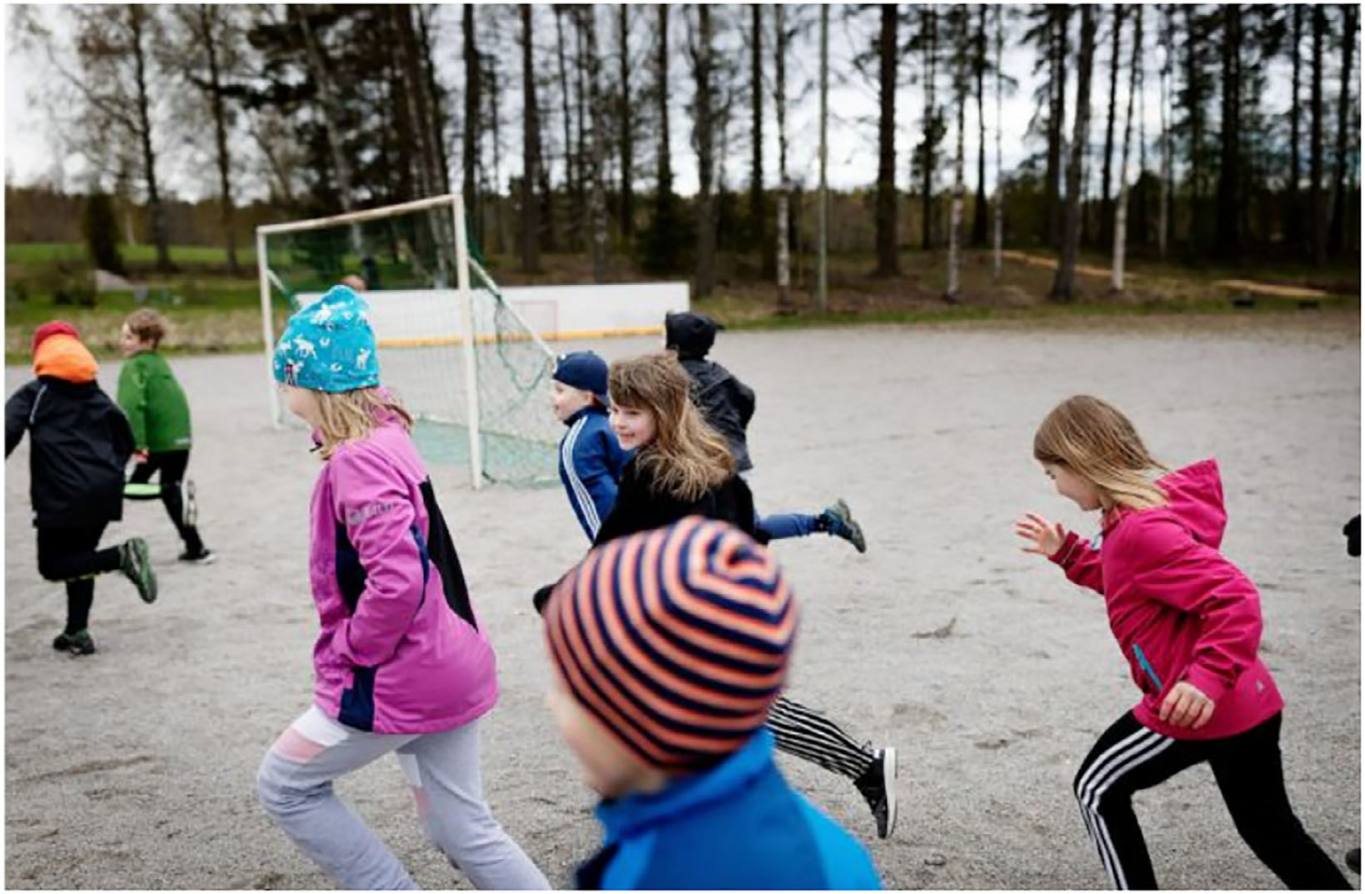
*Helsingin Sanomat* 31.5. 2020. Photo: Sara Aalto

The images of children at school also communicate control. In Figure 4, children are learning how to determine a safe distance using their arms to measure, and Figure 5 shows children standing in a queue with ample distance between one another. An even distance between the children in both photographs creates a regular rhythm communicating control and order. The air of control is fostered through the tight framing and horizontal direction of the image. Figure 6 in turn shows a child washing his/her hands. Once again, the high angle contributes to the air of control. Despite the discipline and order, the return to school appears fun as well. In Figure 7, pupils jump as an expression of joy when meeting each other again after the lockdown, and in Figure 8, the joyful facial expressions of the children playing as well as the free composition of the snapshot image communicate happiness and spontaneity. These kinds of images may be intended to remind the reader that children may easily forget COVID‐19 safety precautions.

### 3.2 Youth: Future‐oriented graduates and reckless partygoers

**FIGURE 9:**
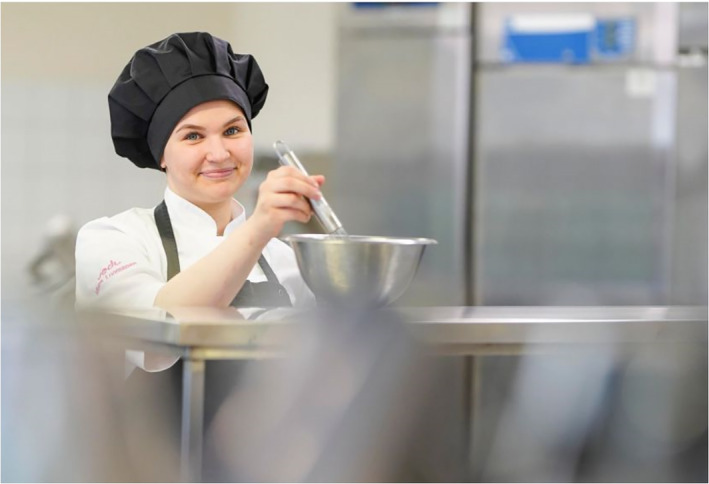
*Helsingin Sanomat* 23.5.2020. Photo: Timo Aalto

**FIGURE 10:**
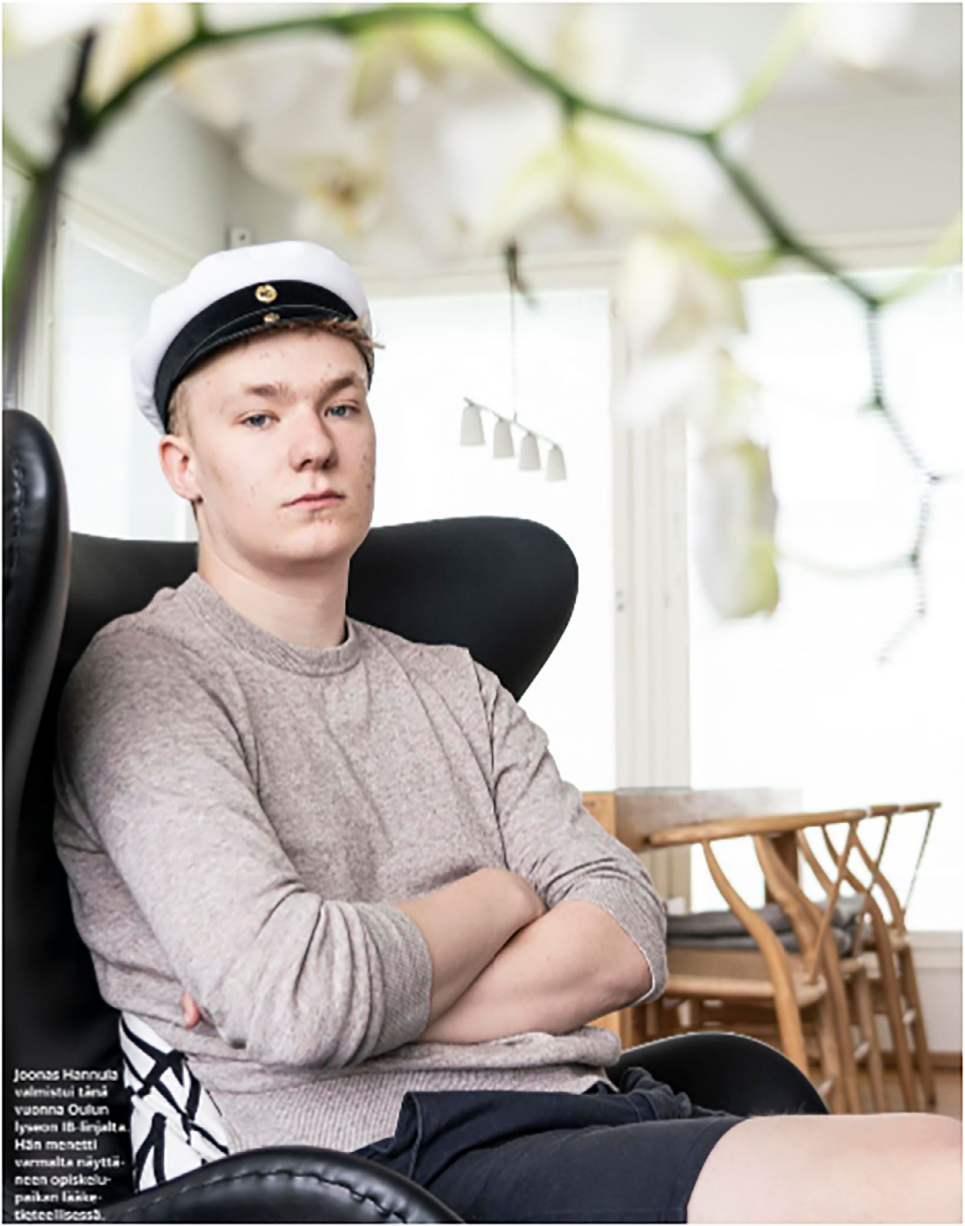
*Helsingin Sanomat* 11.7.2020. Photo: Vesa Ranta

The photographs depicting youth construct for them two principal subject positions, namely future‐oriented graduates and reckless partygoers. A cluster of images depicts youth who have completed or are about to complete their upper secondary education or who are studying to pass higher education entrance examinations. Students about to complete their upper secondary vocational education are shown completing their practical training period in the workplace (Figure 9). The chef's outfit and working/learning environment suggest the female student is about to enter work life. Her facial expression and eye contact with the spectator communicate optimism. Figure 10 shows a male student who has completed his matriculation examination and wears the white matriculation cap as a symbol of this accomplishment. Unlike the high‐angle images of the children, these photos of youth are taken from eye level, which makes them appear equal with the spectator. In fact, the slightly low angle in the image of the male student together with his firm pose and gaze cast straight at the spectator communicate assurance and self‐determination. Both students are positioned in the left panel of the golden ratio with torsos turning to the right, the direction of the future. The location and direction construct an air of optimism and future orientation.

**FIGURE 11:**
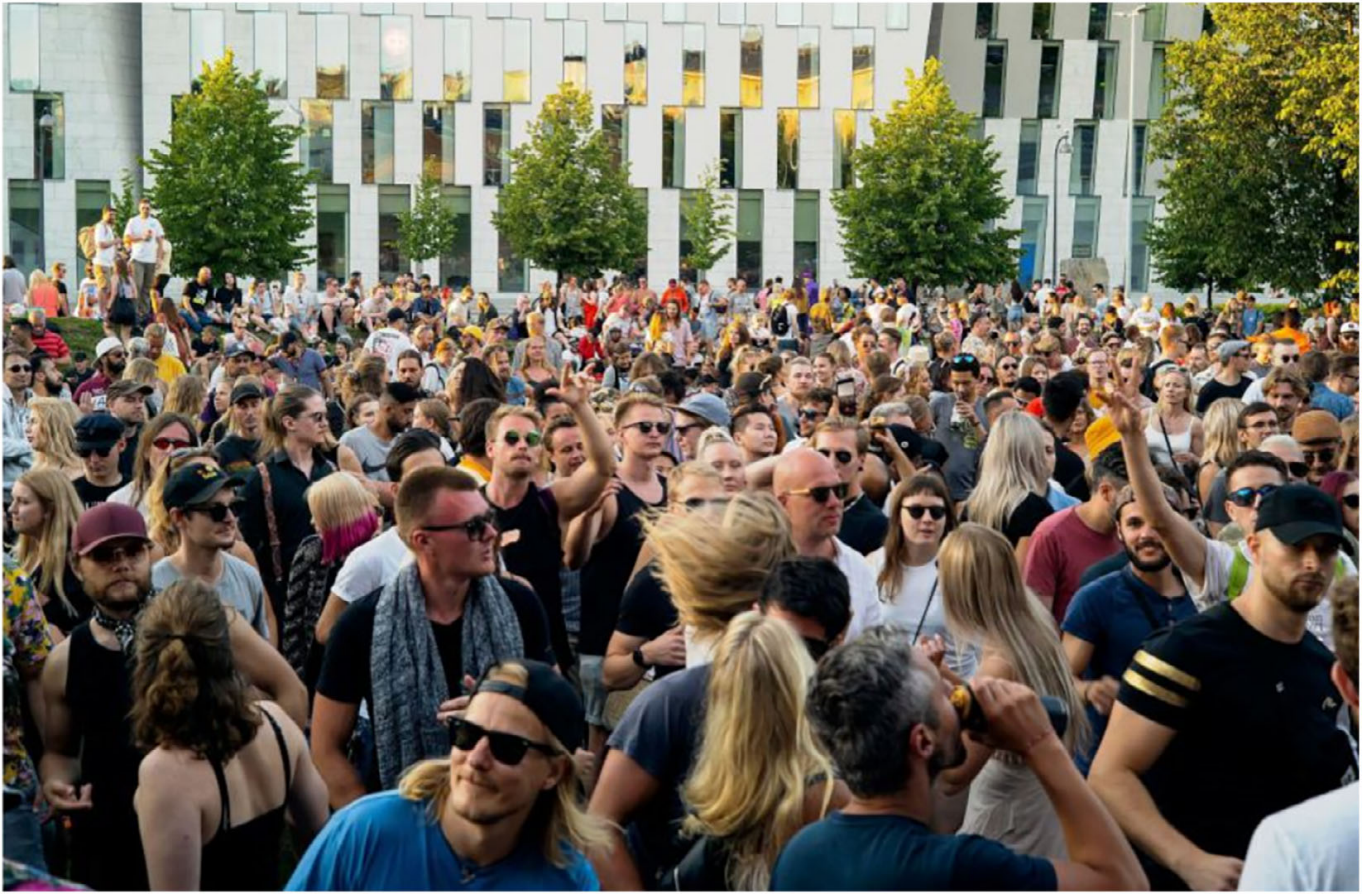
*Helsingin Sanomat* 28.7.2020. Photo: Henri Airo/HS

**FIGURE 12:**
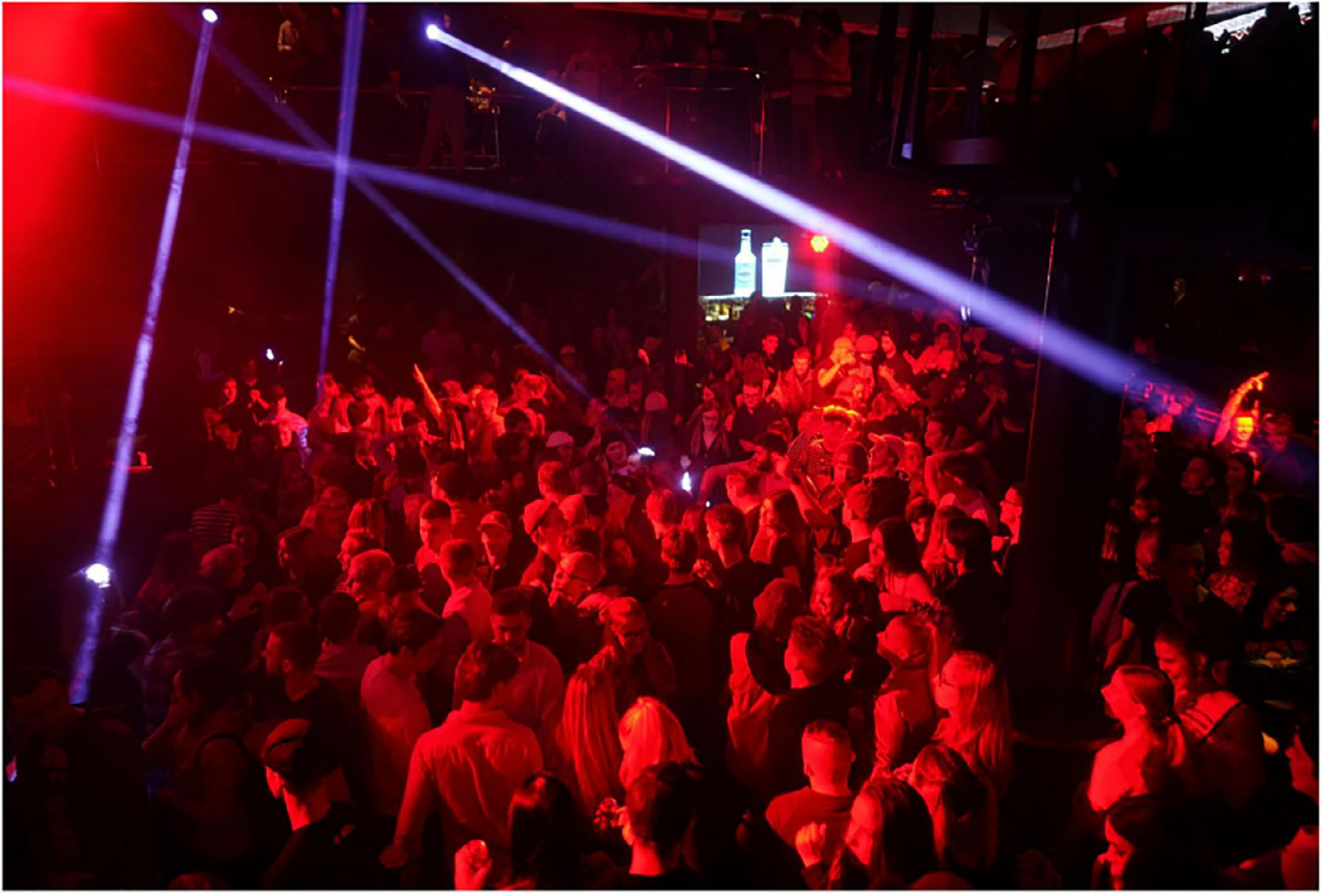
*Helsingin Sanomat* 18.7.2020. Photo: Sami Kero/HS

**FIGURE 13:**
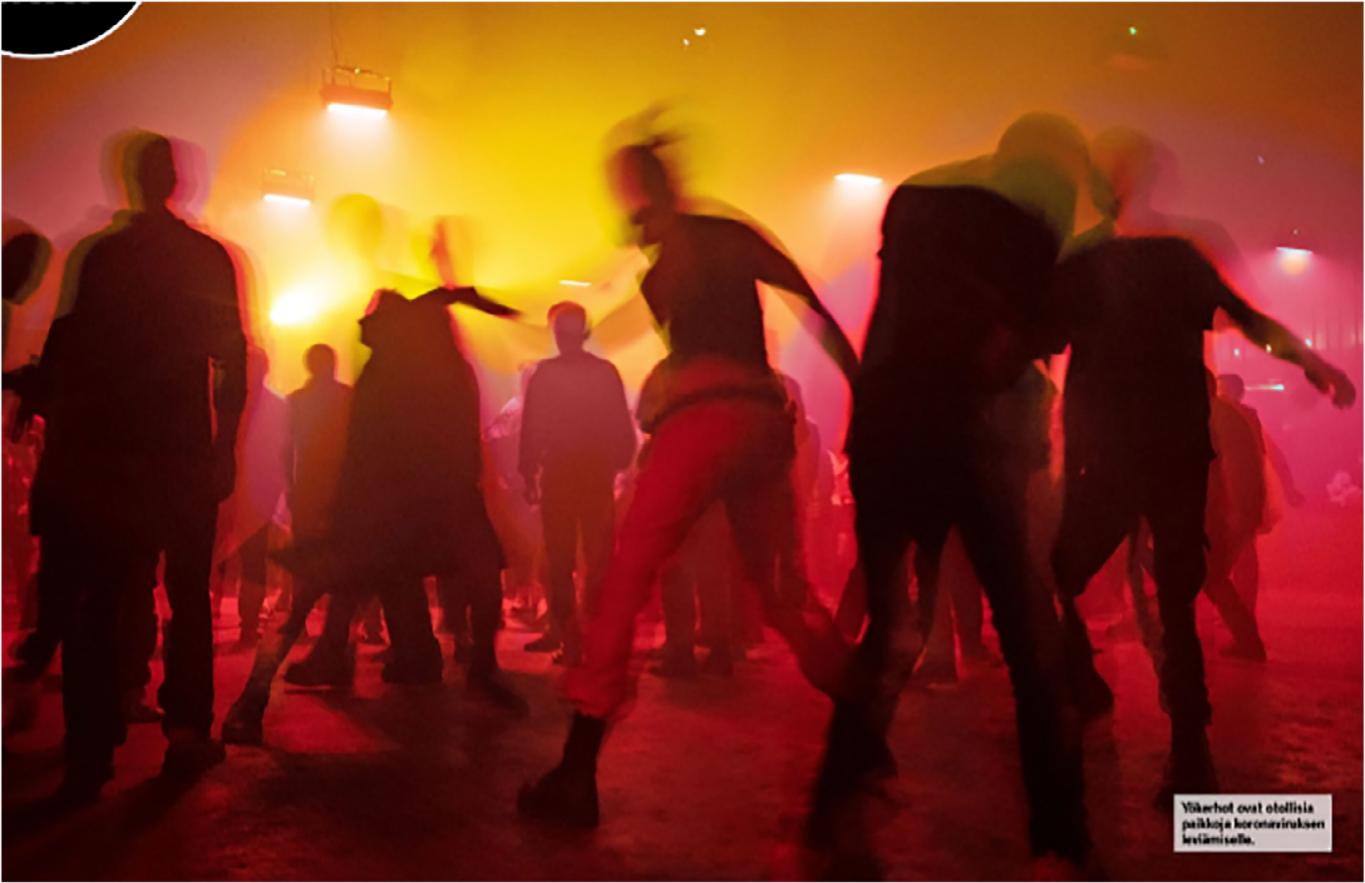
*Ilta‐Sanomat* 18.8.2020. Photo: Outi Pyhäranta

**FIGURE 14:**
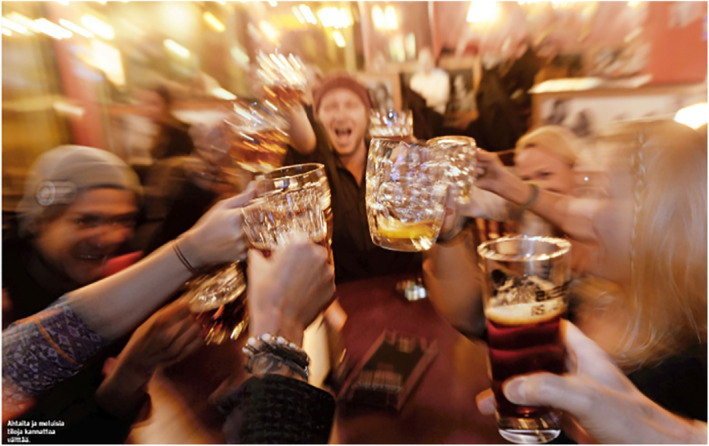
*Ilta‐Sanomat* 28.5.2020. Photo: Vesa Moilanen/Lehtikuva

Another cluster of images depicts youth as irresponsible partygoers who defy the risks of being infected with COVID‐19 or transmitting the virus. Irresponsibility is expressed through masses of young people packed in a festival area or club, a lack of physical distancing and an absence of face masks. In addition, Figures 11, 12 and 13 evoke feelings of restlessness and carelessness through people's movement, colourful lights and unarranged compositions. Images of youth partying in clubs have a moralising overtone constructed through the reddish light often associated with loose morals, liberal sexual behaviour and sin. This moralising overtone is also communicated through images of youth getting drunk. In Figure 14, drunkenness is visualised by blurring the images of the young people. In this subject position, youth are depicted as irresponsible and immoral partygoers whose reckless behaviour threatens the safety/health of society.

In addition to these two most frequent subject positions, some images depict youth travelling, shopping, participating in sports or engaging in other leisure activities.

### 3.3 Adults: Authoritative experts, adaptive professionals, responsible caretakers and active recreationists

**FIGURE 15:**
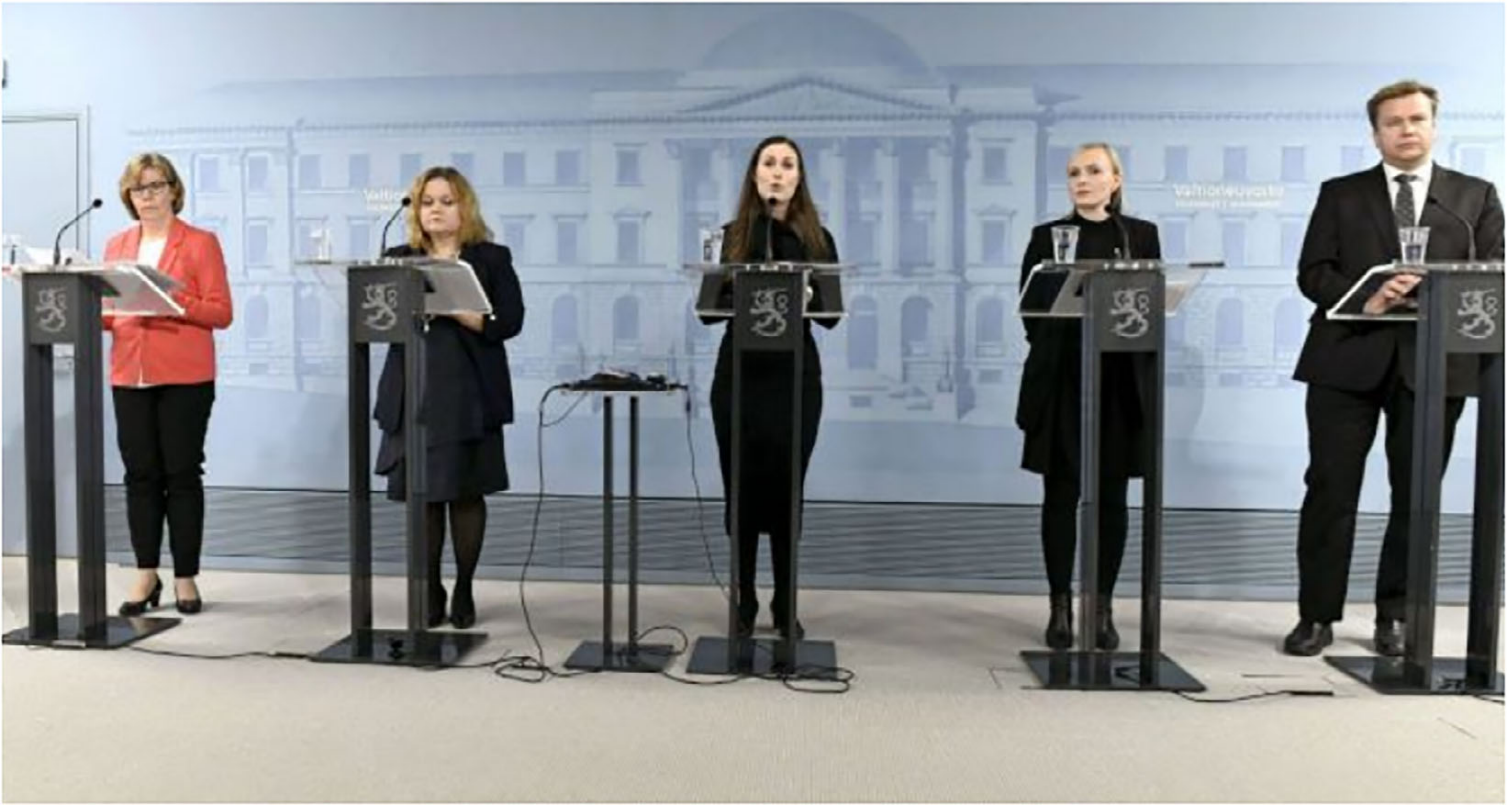
*Ilta‐Sanomat* 1.4.2020. Photo: Heikki Saukkomaa/Lehtikuva

**FIGURE 16:**
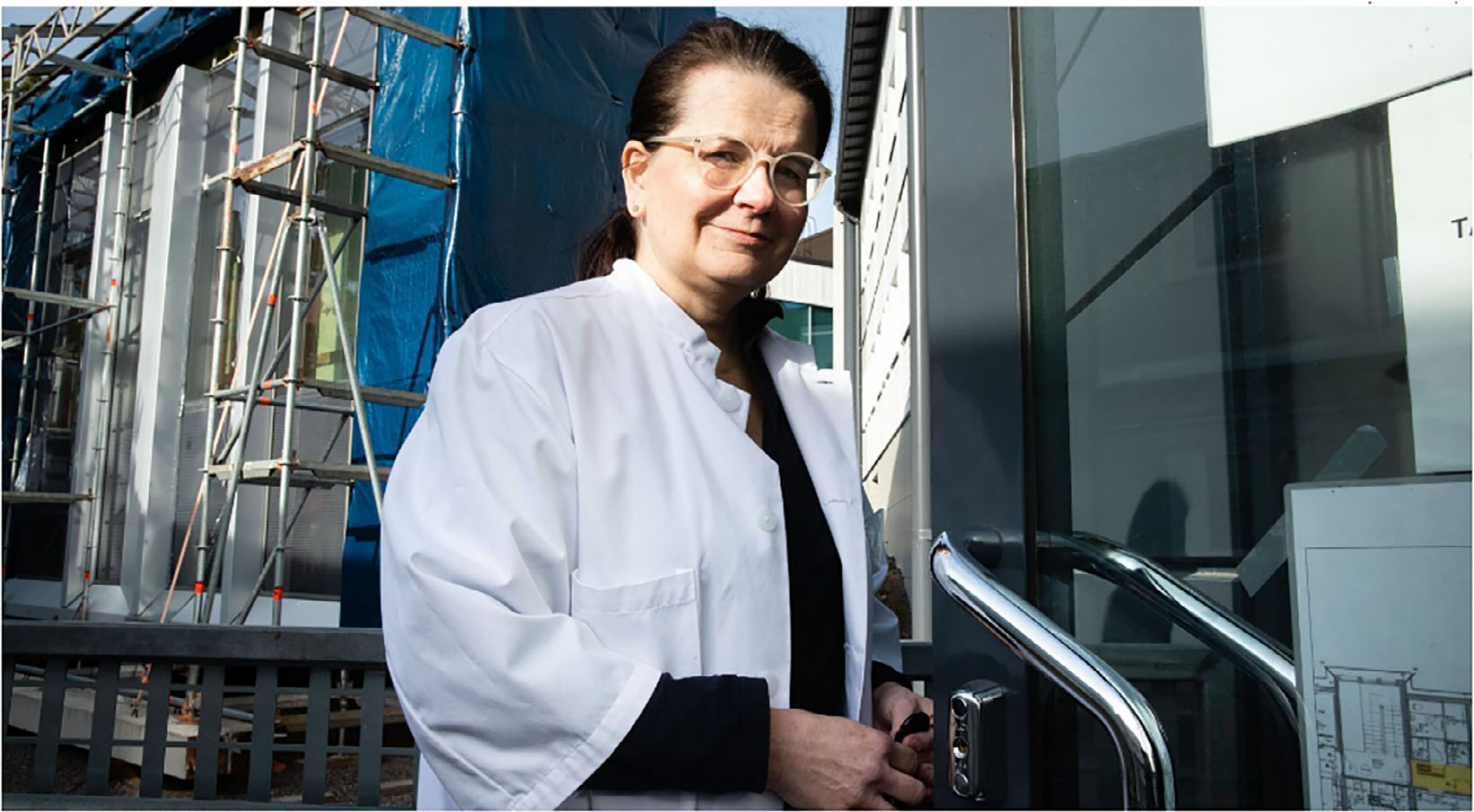
*Ilta‐Sanomat* 9.4.2020. Photo: Juhani Niiranen/HS

**FIGURE 17:**
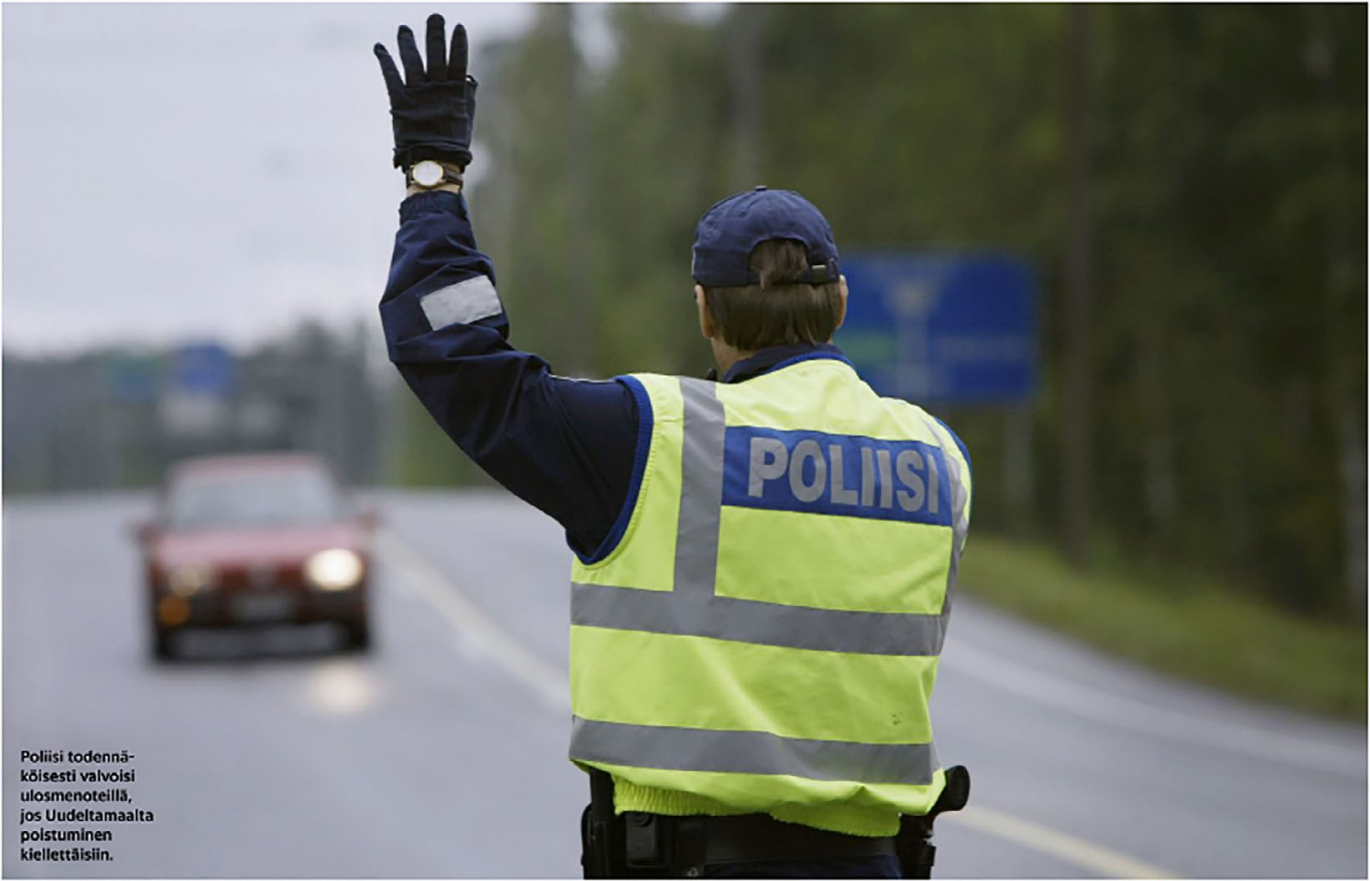
*Helsingin Sanomat* 25.3.2020. Photo: Antti Johansson/HS

The adult age group is depicted with the most diversity. First, adults are assigned the subject position of authorities and experts. The dimension of authority is most clearly present in images of politicians, police and health care and economic specialists. Figure 15 shows one of the many press conferences held by the Finnish government during the pandemic. The grey and blue shades, black stands (with the Coat of Arms of Finland) spaced regularly apart and the image of the House of the Senate on the wall behind the ministers create an austere and official‐looking environment that oozes authority. Apart from the red blazer of the minister of justice, all the ministers wear black, formal clothes. The aspect of authority is further enforced through the slightly low angle of the image. A similar viewing angle is adopted in Figure 16, which portrays a health care specialist. Together with the viewing angle, the white doctor coat and centering of the image form the air of expertise and authority. The dimension of authority is mixed with one of order in Figure 17, in which a police officer stops a car due to travel restrictions. Authority and order are expressed through the officer's position at the centre of the image, uniform, yellow safety vest and gesture (telling the approaching car to stop).

**FIGURE 18:**
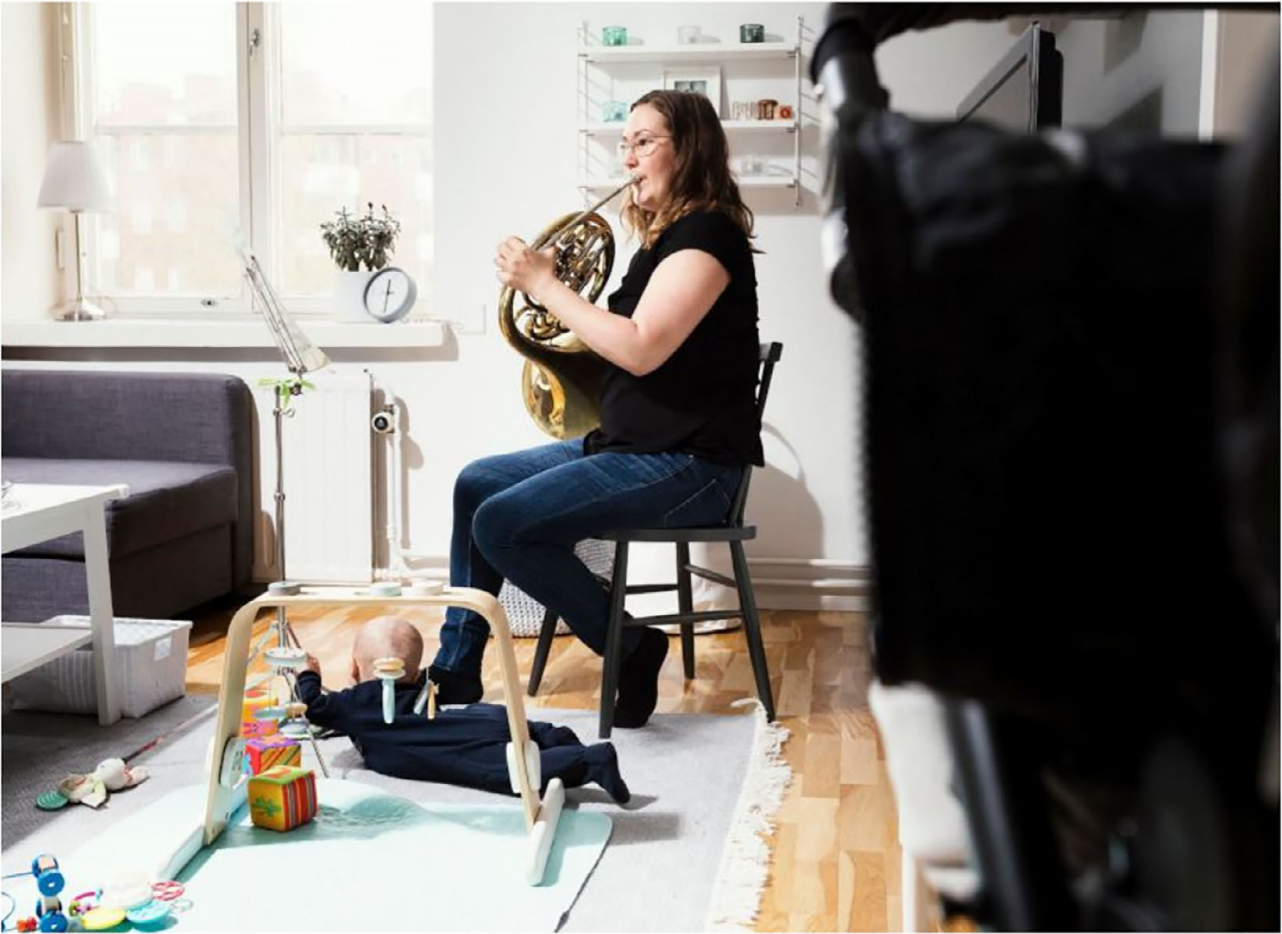
*Helsingin Sanomat* 7.5.2020. Photo: Toivo Heinimäki/HS

**FIGURE 19:**
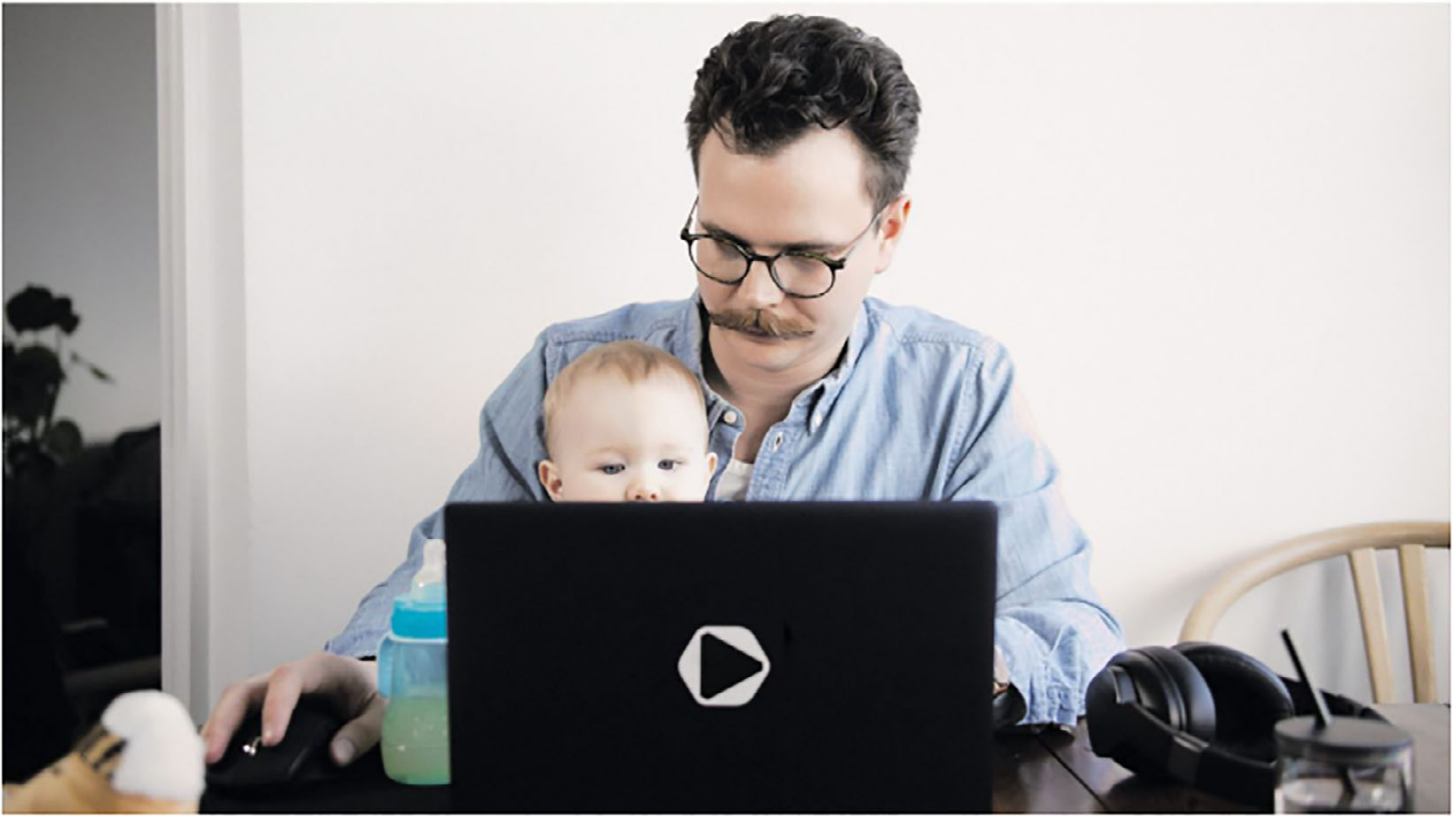
*Ilta‐Sanomat* 9.5.2020. Photo: photographer not mentioned

Adults are also represented as flexible professionals in diverse fields of work (e.g., education, catering, industry, sports and arts and culture) who have had to adapt to distance work at home and quickly develop new practices to fulfil their job duties. Simultaneously, kindergartens and schools were locked down, which resulted in many parents juggling a household, children and a job. In addition, flexible professionals had to follow safety measures (e.g., social distancing and wearing face masks), adapt to continuously changing conditions and cope with uncertainty regarding their future employment. Visually, these adaptive multitaskers are represented through images in which they perform multiple chores simultaneously or complete tasks online that were once done face to face. In Figure 18, a music teacher teaches the French horn online while simultaneously looking after the baby on the floor. A similar scene is depicted in Figure 19, where a father is working on a laptop and holding a baby on his lap.

**FIGURE 20:**
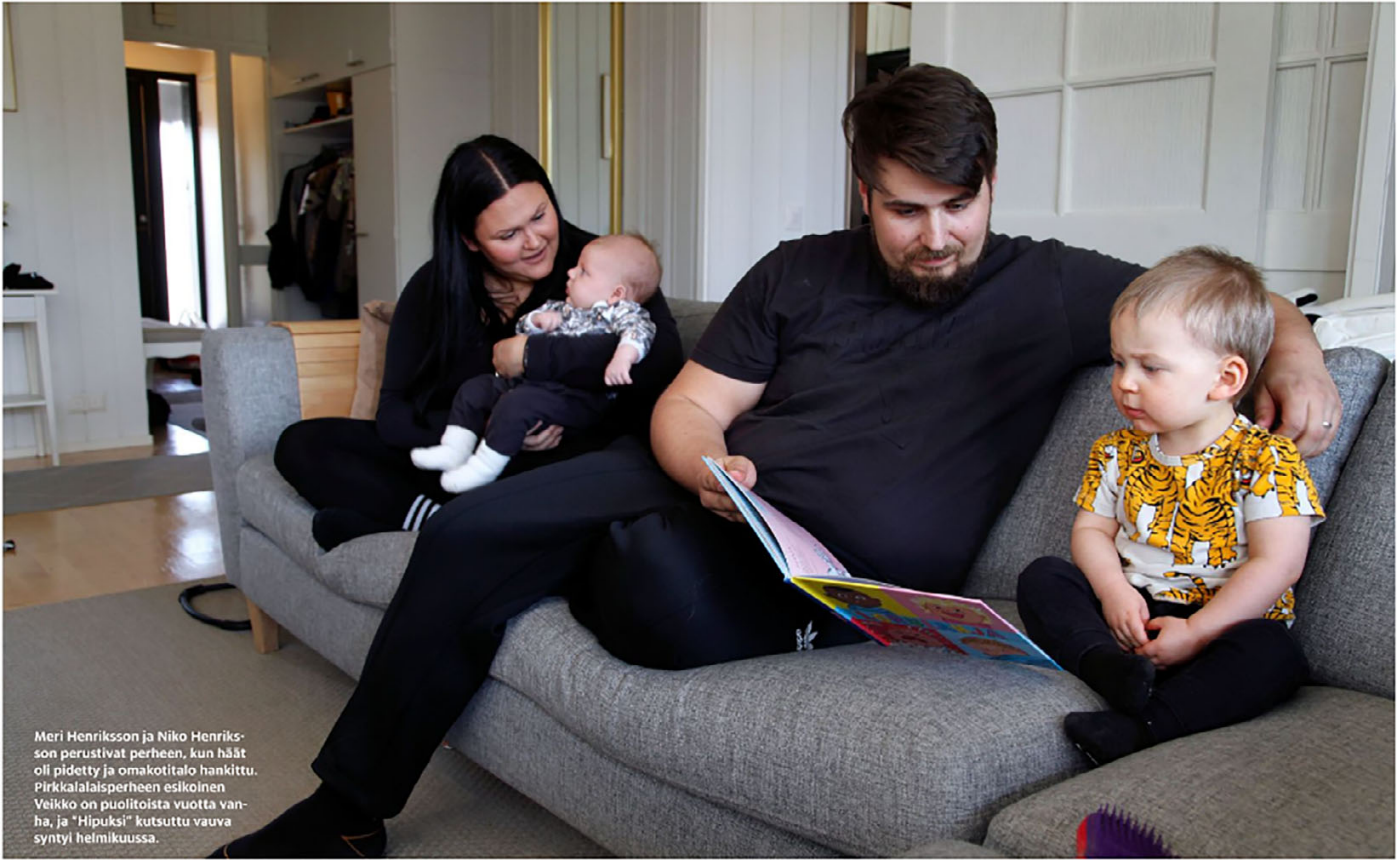
*Helsingin Sanomat* 10.4.2020. Photo: Reijo Hietanen

**FIGURE 21:**
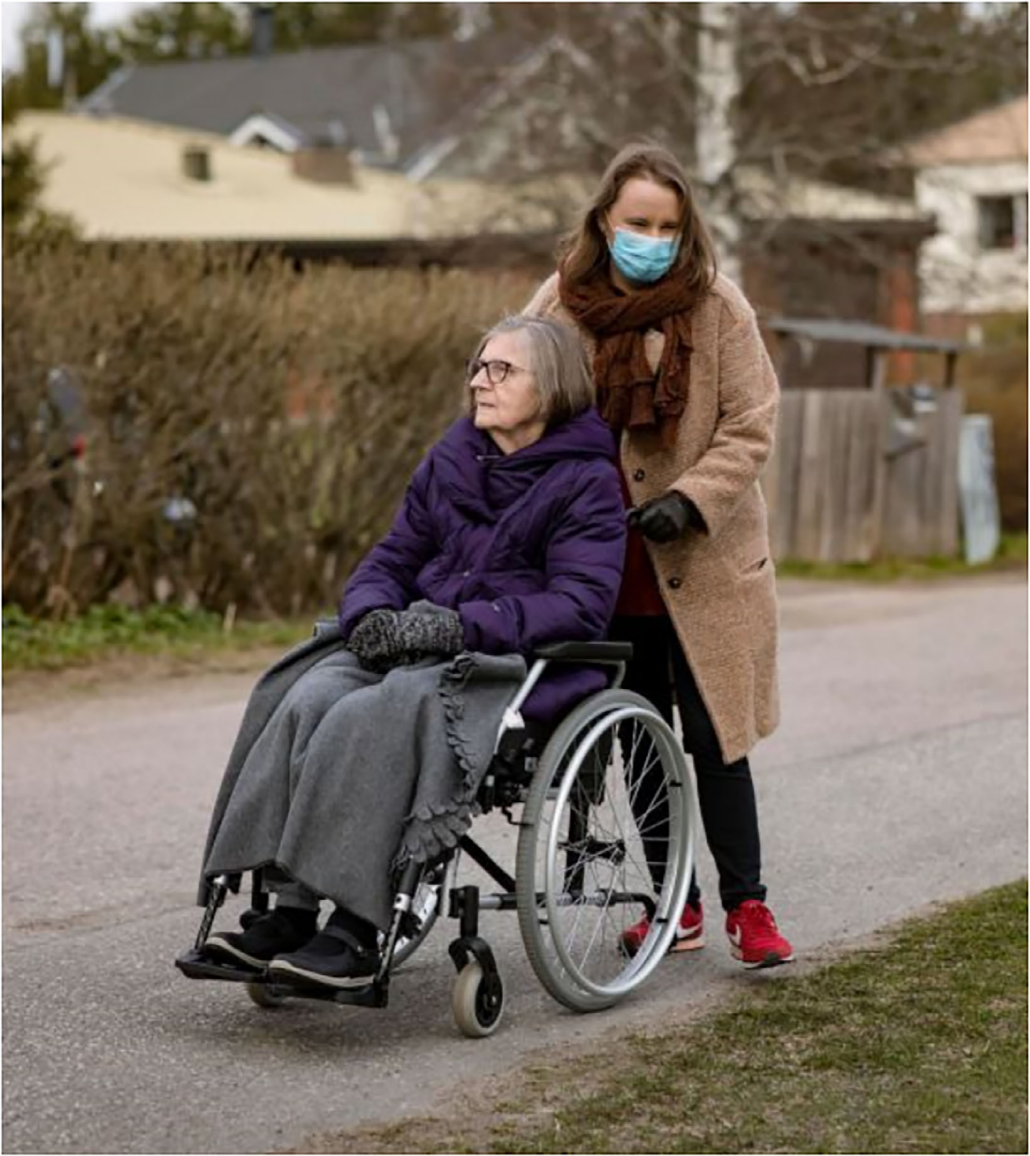
*Helsingin Sanomat* 8.5.2020. Photo: Mika Ranta/HS

**FIGURE 22:**
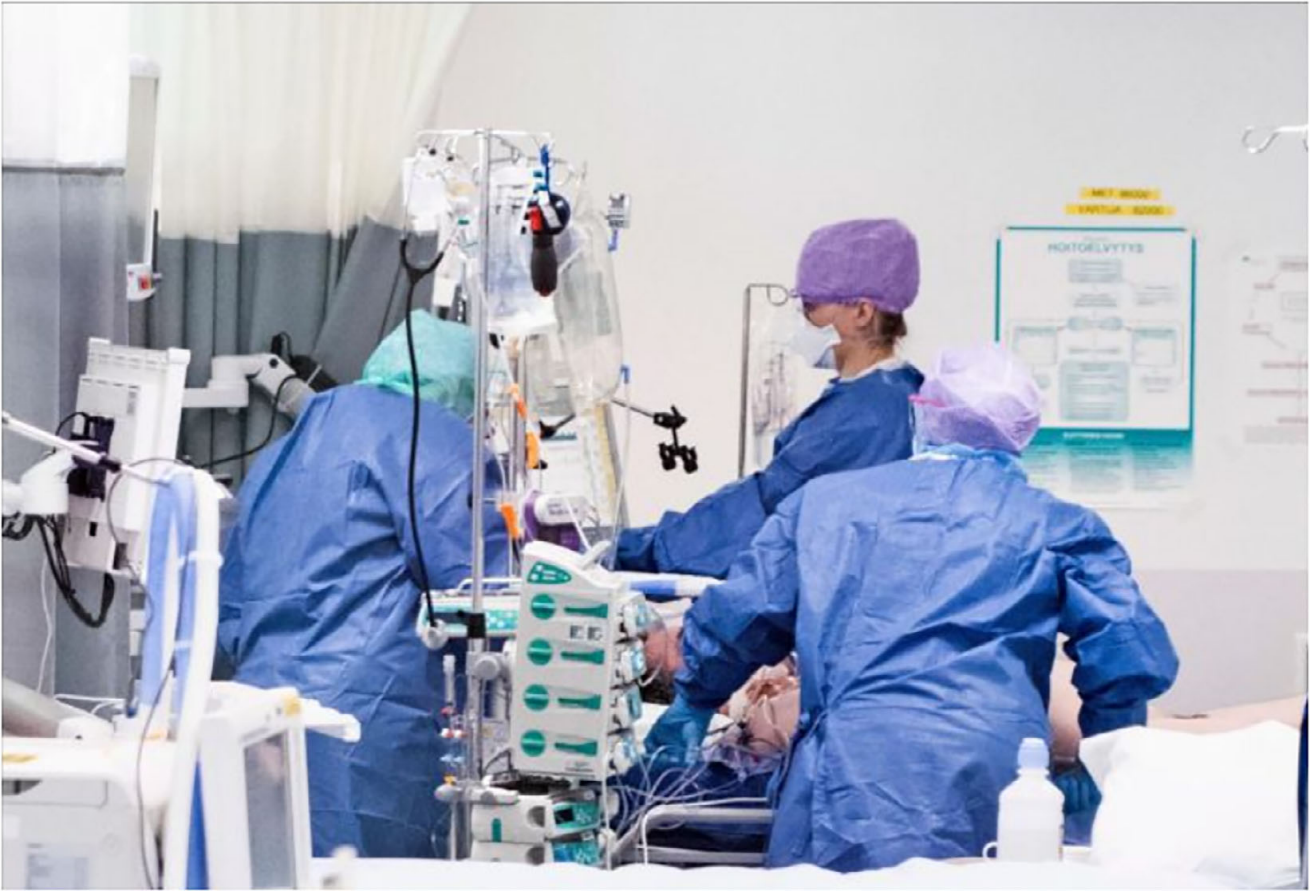
*Ilta‐Sanomat* 17.6.2020. Photo: Pietari Hatanpää

**FIGURE 23:**
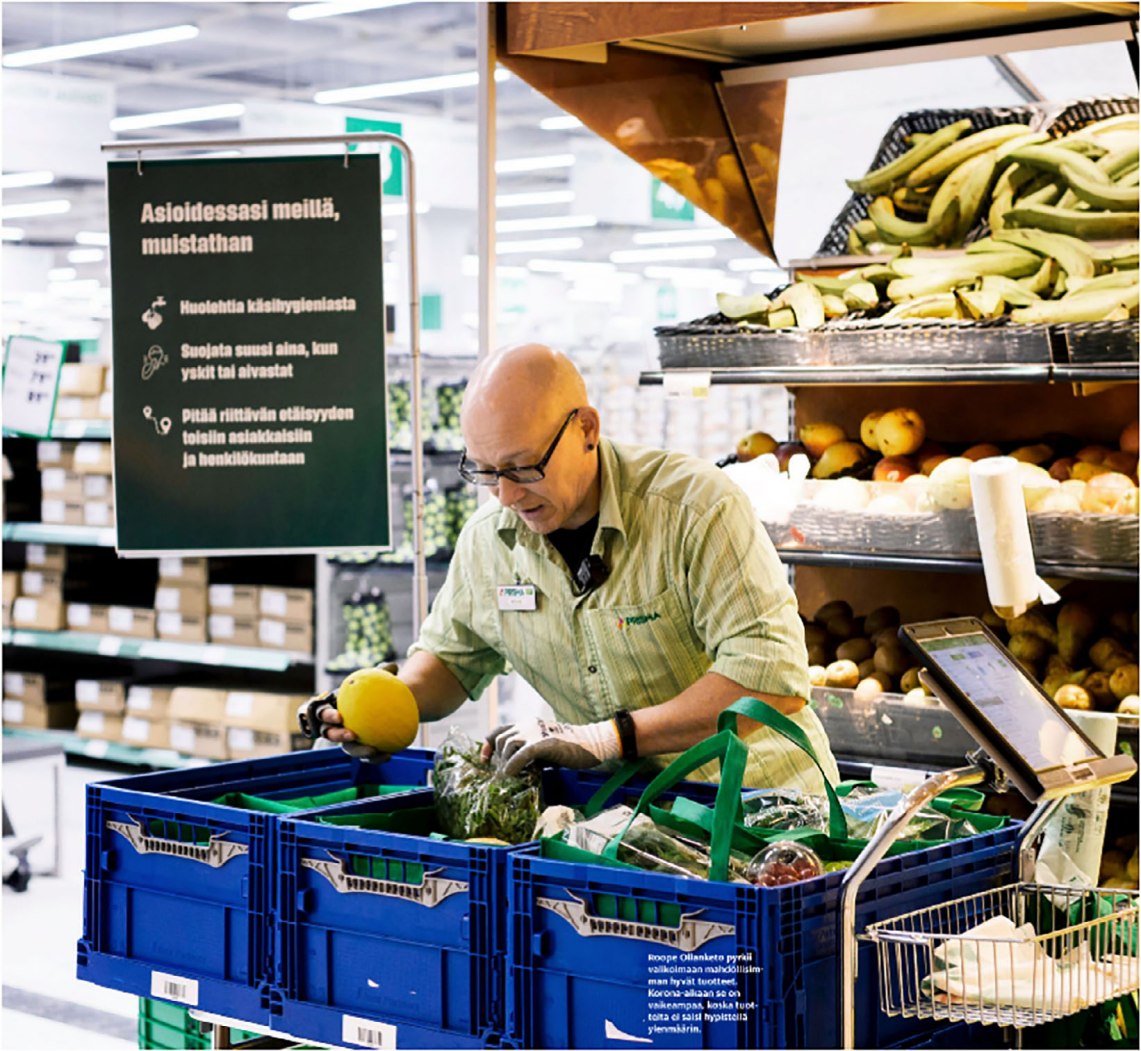
*Helsingin Sanomat* 25.4.2020. Photo: Toivo Heinimäki/HS

The third subject position for adults depicts them as responsible caretakers of the other three age groups discussed in this study. This depiction includes health care professionals caring for those infected with COVID‐19. The aspect of care is visualised through bodily contact and touch (Figure 20), the concrete act of helping (Figure 21) and professional care provided in hospitals (Figure 22). This group of caretakers may also include shopkeepers and volunteers who deliver food to elderly and sick people in quarantine (Figure 23). The aspects of affection, help and solidarity are identifiable in these images.

**FIGURE 24:**
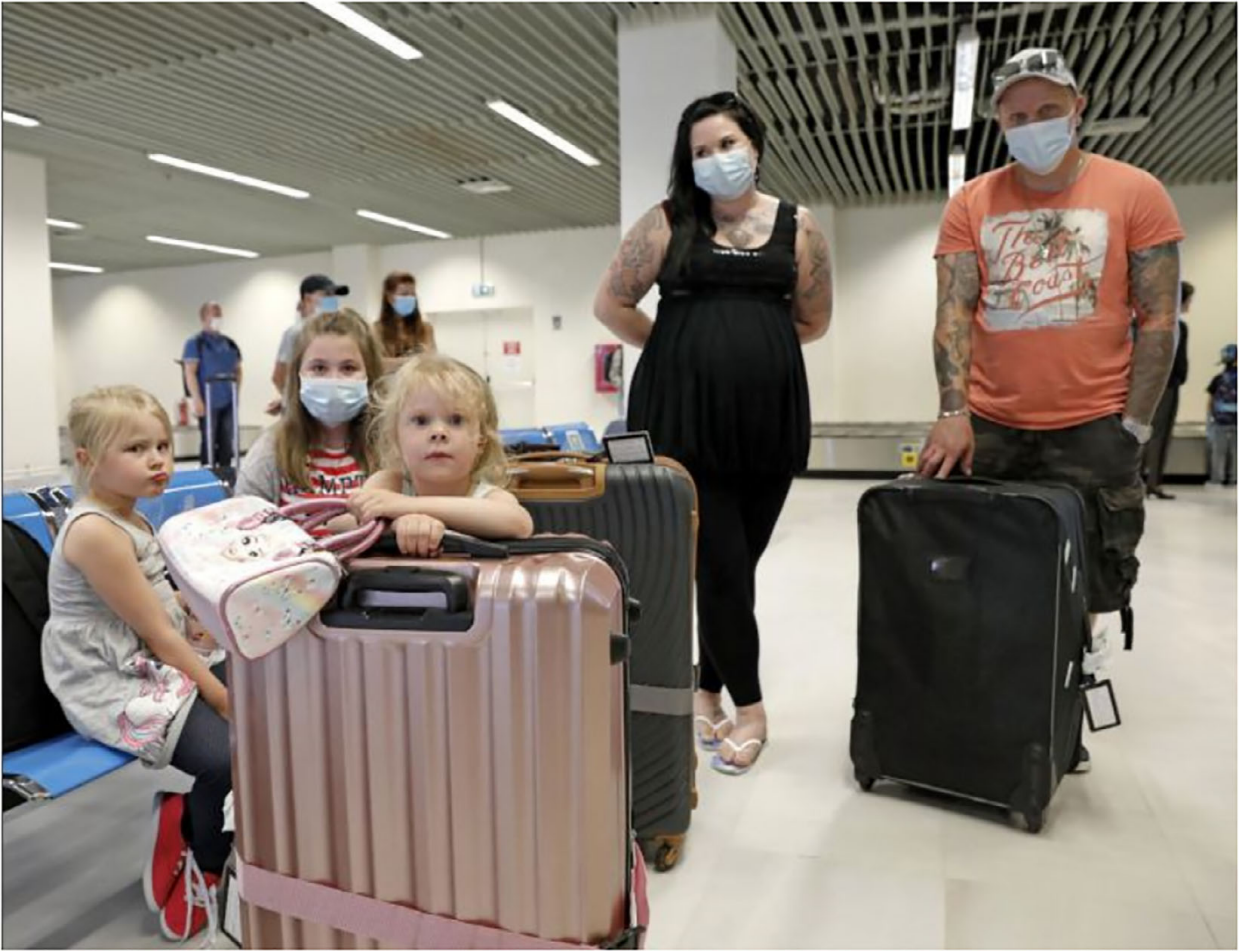
*Ilta‐Sanomat* 17.7.2020. Photo: Antti Hämäläinen/IS

**FIGURE 25:**
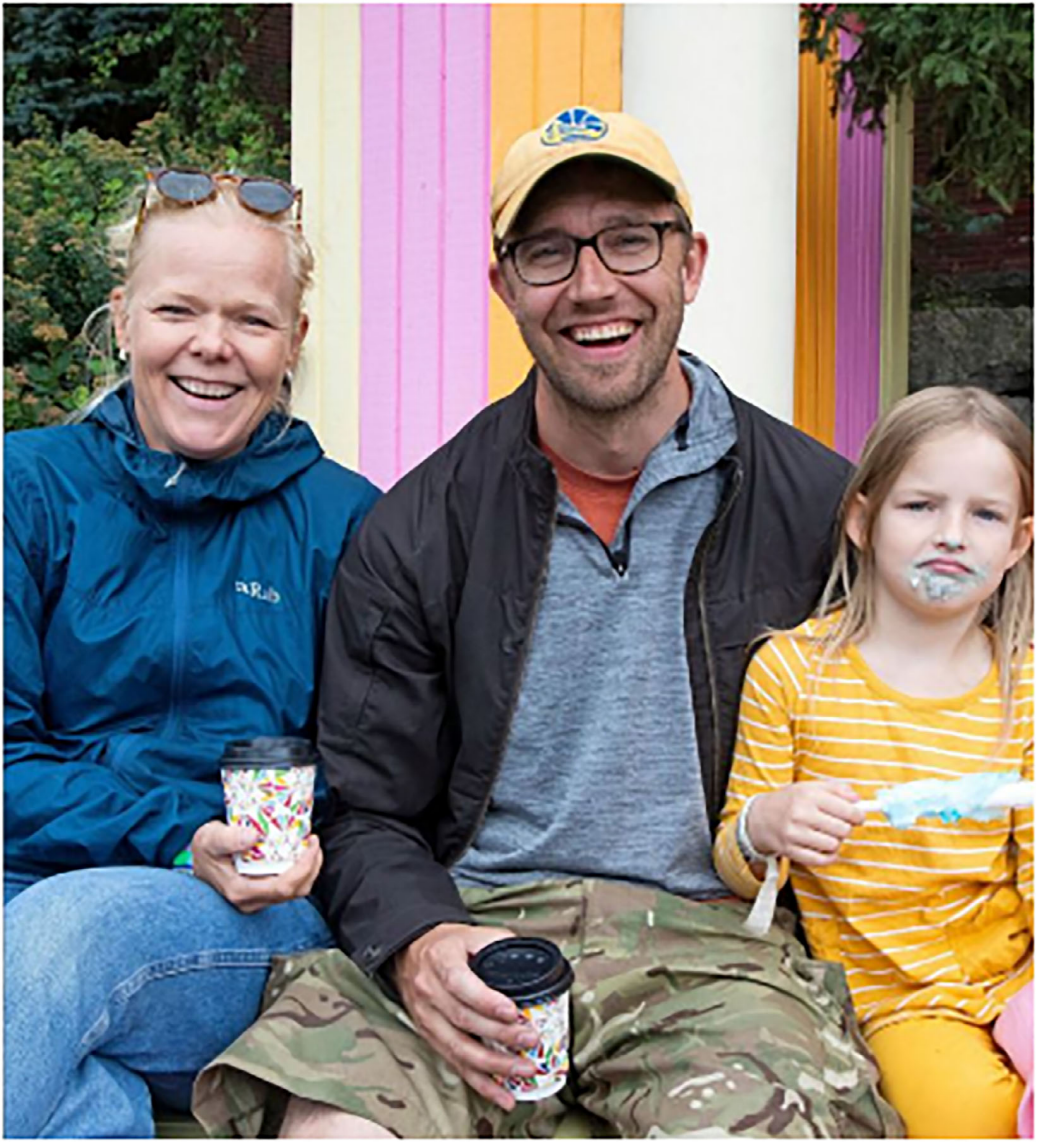
*Ilta‐Sanomat* 24.7.2020. Photo: Aleksi Jalava/IS

**FIGURE 26:**
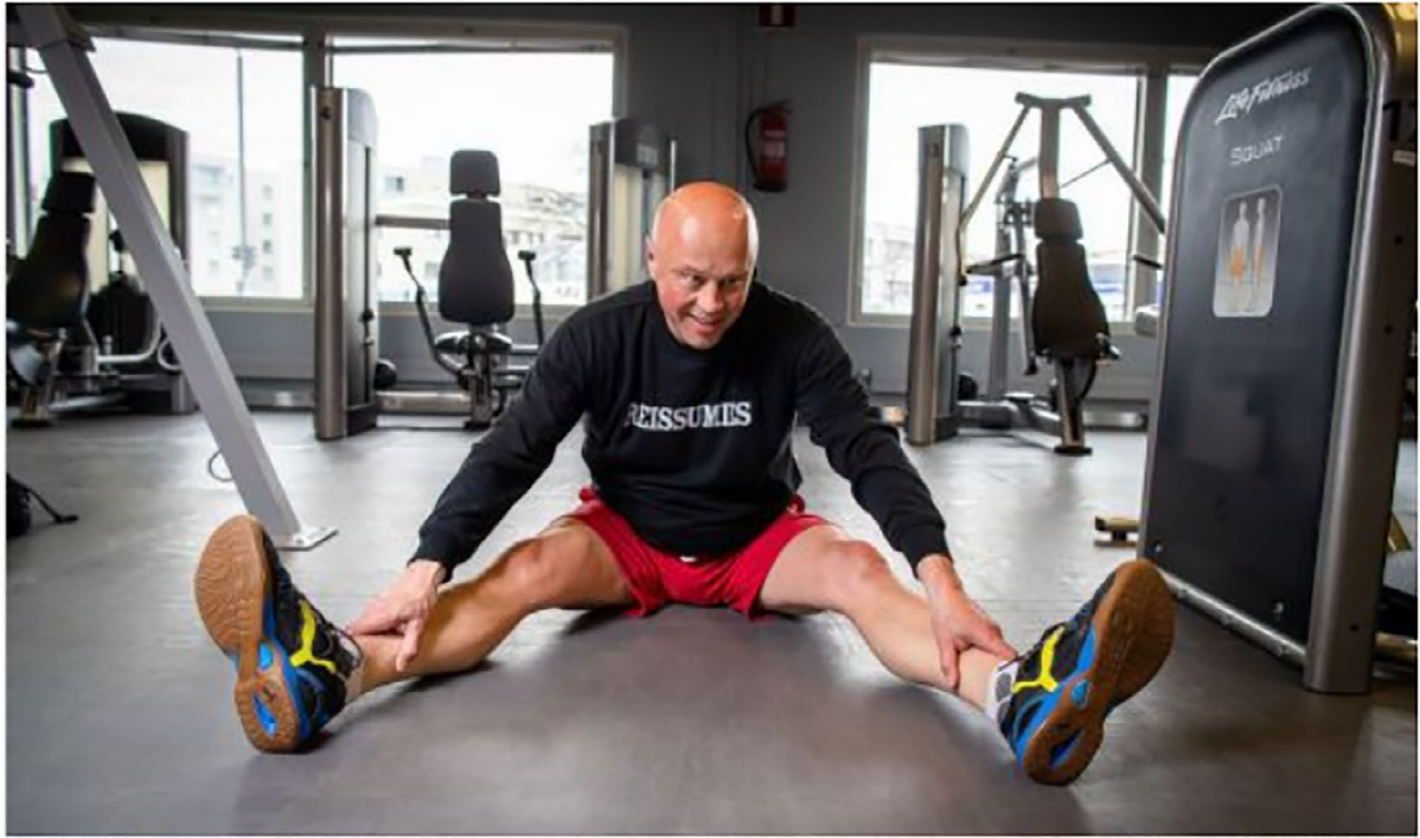
*Ilta‐Sanomat* 25.4.2020. Photo: Kalle Koponen/HS

The fourth subject position is adults as active recreationists. These images portray adults enjoying free time and leisure activities alone or with their families. Relaxation is visually depicted through casual clothing, smiling faces, travel bags or carefree moments (no chores or job duties being performed; Figures 24, 25 and 26). Typical imagery of active recreationists shows adults exercising at home, the gym or outdoors. The captions often mention that sports help with managing stress.

In addition to the aforementioned four most frequent subject positions of adult people, the data included a number of images of ordinary Finnish people and celebrities who have expressed their views on diverse matters related to COVID‐19, talked about their lives during the pandemic or shared their experiences after having been infected with the virus. Furthermore, some images depict marginalised groups of people, such as refugees or homeless people.

### 3.4 Elderly people: Isolated loners

The fourth group of people, the elderly, are the most uniformly depicted in the photographs. In both newspapers, elderly people are sometimes depicted as sick with COVID‐19 or exercising outdoors. In addition, some images feature famous elderly Finnish people discussing their lives in quarantine. However, the majority of the photographs depict elderly people in isolation, either in nursing homes or their own homes.

**FIGURE 27:**
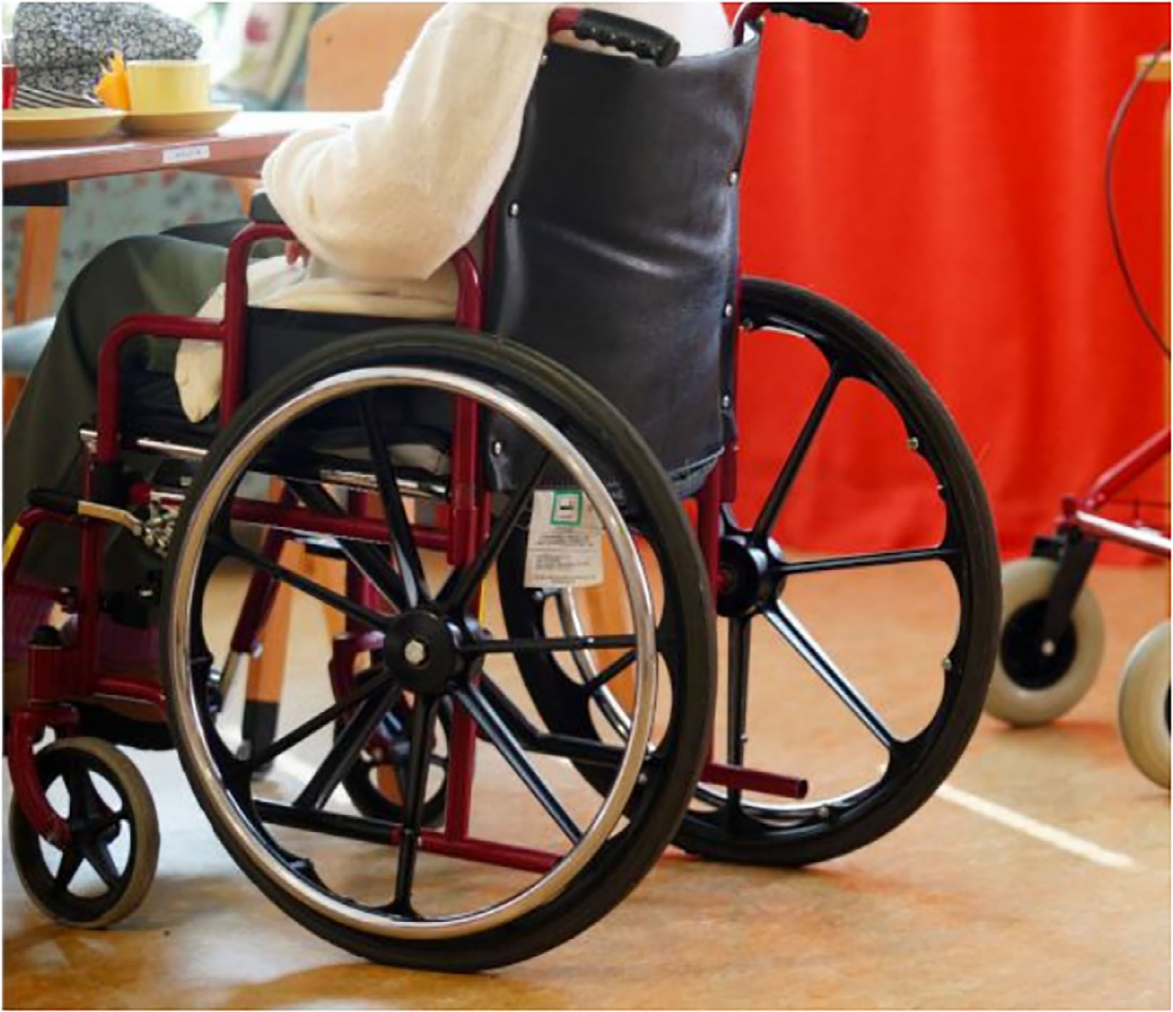
*Helsingin Sanomat* 10.4.2020. Photo: Aleksi Kinnunen

**FIGURE 28:**
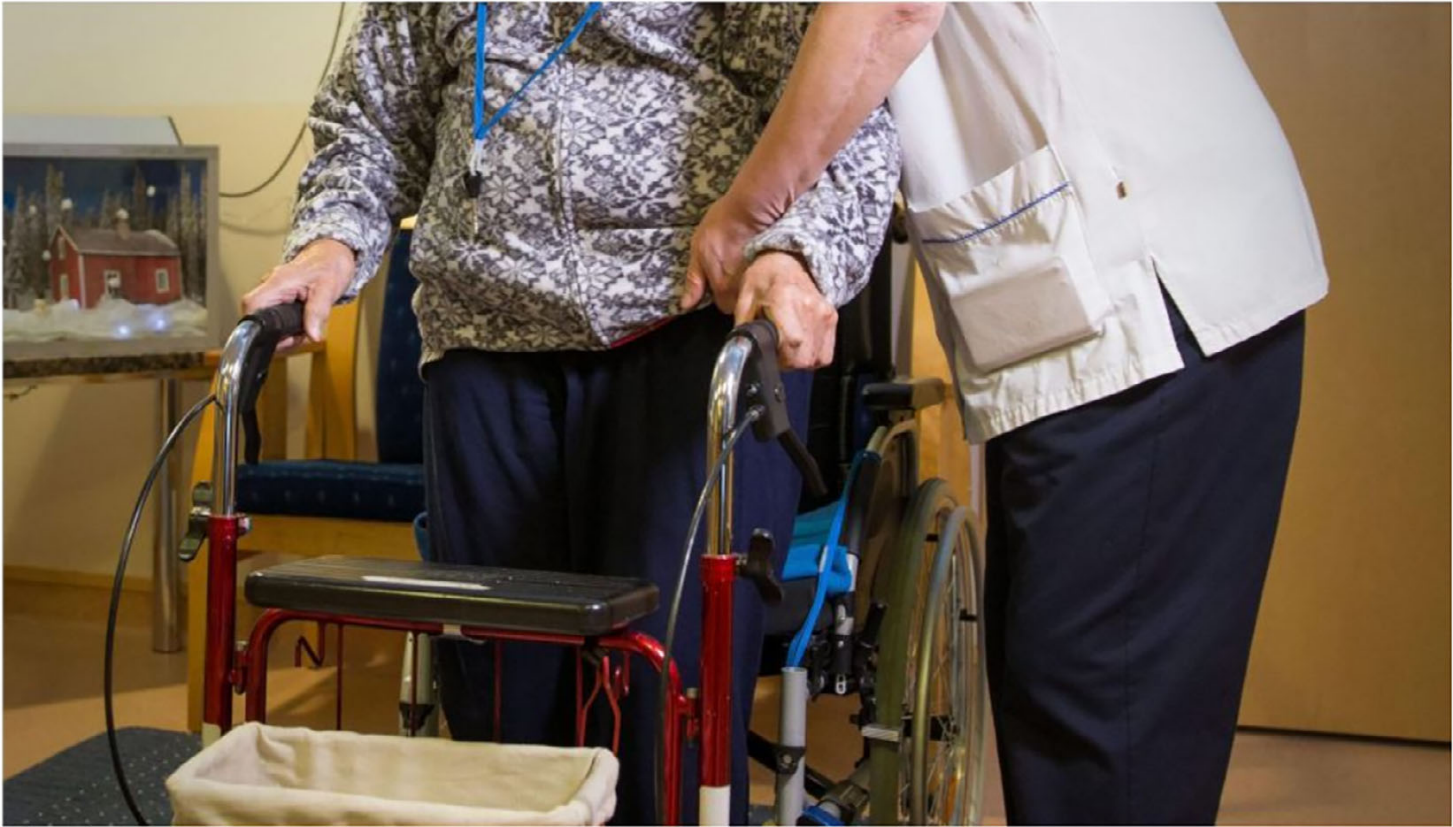
*Helsingin Sanomat* 28.4.2020. Photo: Jarmo Sipilä

In some nursing homes in Finland, inhabitants were infected with COVID‐19, leading to numerous deaths. This led to strict regulations and precautions in nursing homes in order to prevent similar incidents. Elderly people's contact with others was minimised, relatives were not allowed to visit the homes, and their outdoor activities were restricted. The rhetorical strategies of the photographs visualise the situation painstakingly. Elderly people are mainly depicted sitting indoors in wheelchairs, lying in bed or walking down corridors with the support of a nurse. Generally, their faces are not shown; instead, only hands and legs/feet are visible (Figures 27 and 28). Although the absence of faces could be for ethical reasons/permissions, this depiction deindividualises (and dehumanises) the subjects.

**FIGURE 29:**
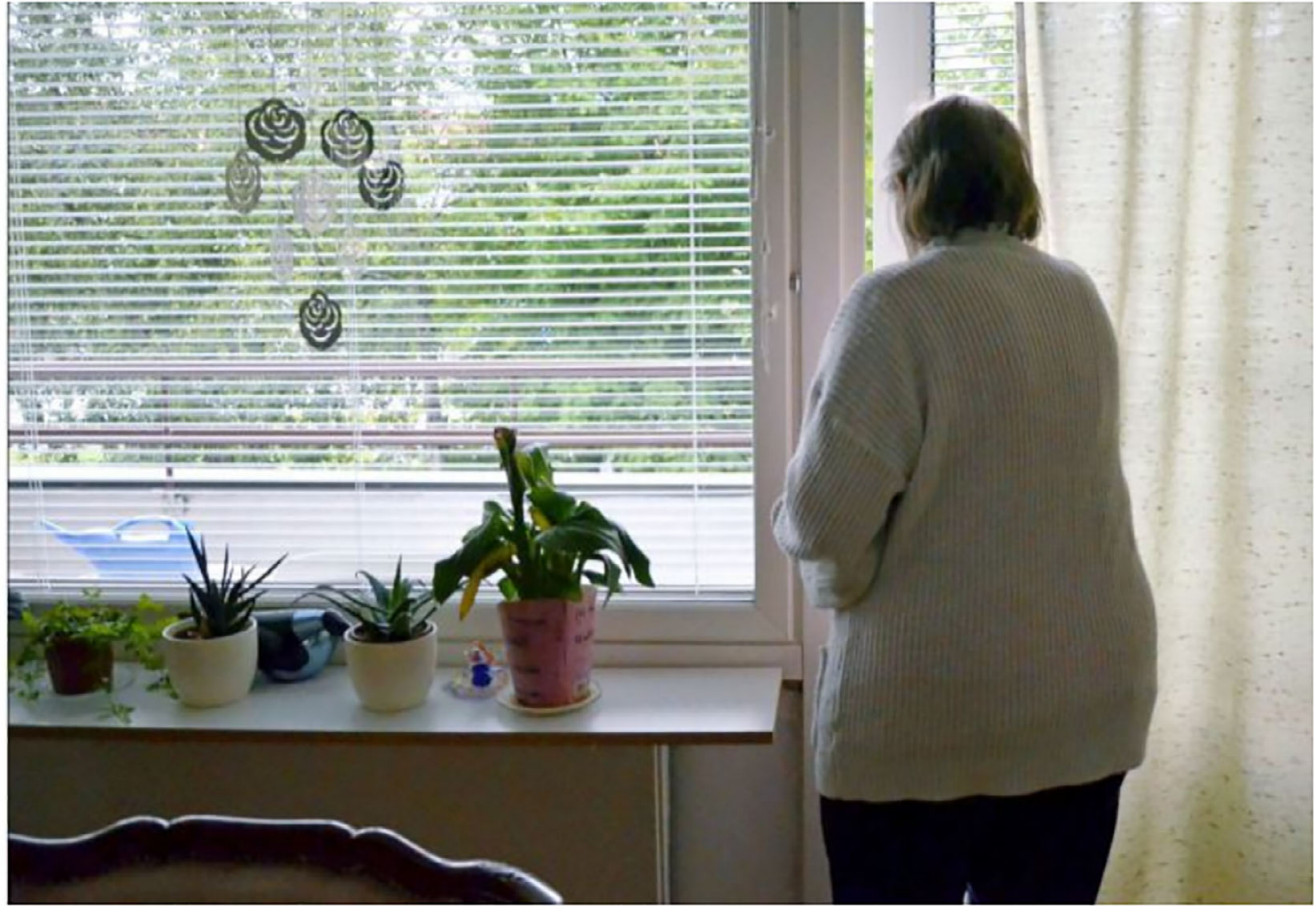
*Ilta‐Sanomat* 19.3.2020. Photo: Eija Kontio/Lehtikuva

**FIGURE 30:**
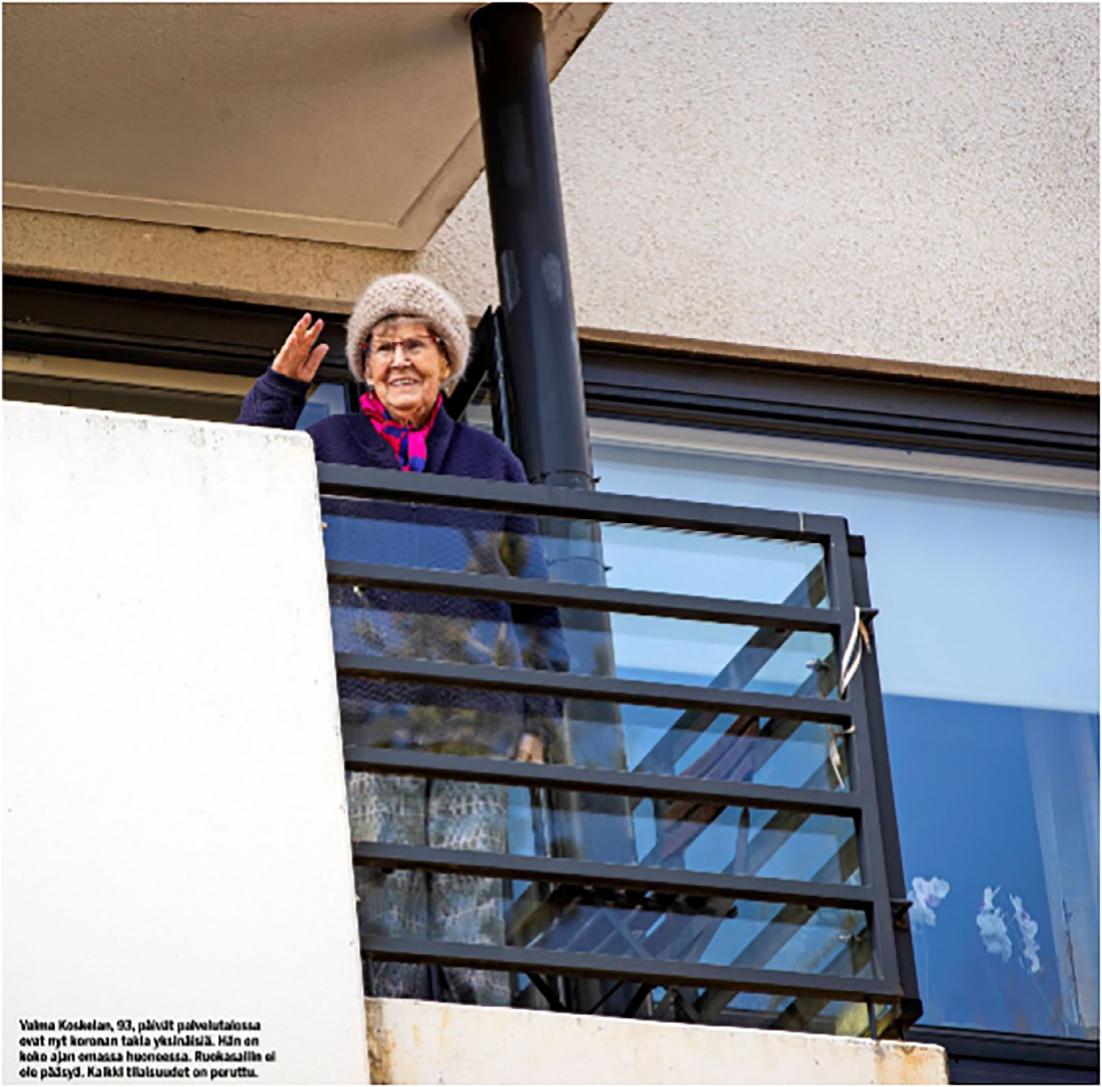
*Ilta‐Sanomat* 2.5.2020. Photo: Antti Hämäläinen

The photos depicting elderly people in their own homes frequently show seniors looking out through a window or waving their hands to their friends or children from behind the rails of a balcony (Figures 29 and 30). In these images, the window screen and balcony rails form physical barriers that imprison the elderly in their homes and separate them from the outside world. In addition, the grey tones of the images and the fact that the person is shown alone construct an air of loneliness and sadness. Images of elderly people communicating with their children or grandchildren through a window visualise the longing for touch, which headlines and captions in both newspapers frequently highlighted. The images of famous elderly Finnish people sharing their experiences of quarantine clearly differ from other images: they smile and pose for the photographer, for instance.

Table 2 concludes the visual construction of subject positions for different age groups. It summarises the visual strategies used to construct the subject positions as well as their rhetorical functions.

**TABLE 2:** Subject positions, visual strategies and rhetorical functions used in the images of different age groups

Age group	Subject position	Examples of visual strategies	Rhetorical function
Children (*n* = 193)	Controlled pupils (*n* = 114)	Alone Serious facial expressions Engaged in learning High angles Arranged compositions Regular rhythm Tight framing	Control
	Joyful, carefree players (*n* = 79)	Several children Smiling, shouting Engaged in playing Snapshots Unarranged compositions	Life continues on
Youth (*n* = 187)	Future‐oriented graduates (*n* = 63)	Matriculation caps Signs of profession Eye level Eye contact Golden ratio Facing to the right	Hope for the future
	Reckless partygoers (*n* = 101)	Crowds of young people Festival/club settings No physical distancing No face masks Reddish light Alcoholic drinks Unarranged compositions	Health risks Moralising Blame
	Depictions of youth engaged in diverse leisure activities (*n* = 23)	Engaged in leisure activities Participating in sports Several young people Smiling	Ease Leisure
Adults (*n* = 3,754)	Authorities and experts (*n* = 1,331)	Austere environments Emblems of state Formal, black clothes Uniforms Restrained gestures Low angles Cold colours	Authority Expertise Power
	Adaptive professionals (*n* = 1,195)	People at work Multitasking Remote work Unusual working conditions	Creativity Flexibility Persistence
	Responsible caretakers (*n* = 453)	Physical closeness Friendly faces Warm colours	Solidarity Heroism Responsibility
	Active recreationists (*n* = 323)	Smiling faces Casual clothes Engaged in leisure activities Participating in sports	Recreation Relaxation
	Depictions of adults sharing their views on and experiences of the pandemic (*n* = 452)	Close‐ups or half‐length images Posing for the photographer Diverse backgrounds	Sharing personal experiences
Elderly people (*n* = 189)	Isolated loners (*n* = 189)	Alone Indoors (mostly) Outdoors (seldomly) Behind windows, balcony rails Grey colours Faces not shown (in nursing homes) Diverse facial expressions and backgrounds (in images of celebrities)	Isolation Loneliness

## 4 DISCUSSION AND CONCLUSION

The aim of this article was to explore social representations and identities related to COVID‐19 in mass media. We focussed on visualisations of COVID‐19 during the first 8 months of the pandemic. To the best of our knowledge, this is the first empirical study analysing media representations related to COVID‐19 over an extended time period. In addition, our focus on images and their visual rhetoric provides a novel perspective on the topic and advances social representations research more broadly. However, there are some limitations to our study. First, it focusses only on media representations of COVID‐19 and not on their perception by the readership. Second, the data were collected from the two largest Finnish newspapers only, and thus, no comparative analysis in relation to media representations in other countries could be made. Finally, the focus on age groups directed the exploration of the subject positions.

Mass media is an important channel of social representation in society, particularly regarding issues that are new and threatening and removed from direct experience (Wagner, Kronberger, & Seifert, 2002). Media cultivate particular understandings of a phenomenon by representing it in a certain way and through meta‐representations. In other words, we rely not only on our own construction of the disease but on how we believe others think about it (Elcheroth et al., 2011). The massive volume of COVID‐19‐related photographs in the media suggests that visualisation has become a key method for news and other information outlets to capture public attention. Visual imagery concretises the threat by providing viewers with tangible and emotion‐evoking examples that act as visual proof. In line with Smith and Joffe's (2013) study on visualisations of global warming, it is reasonable to believe that although various depictions of COVID‐19 are circulating in the public sphere, it is the visual material that has the greatest impact on public engagement.

The visual rhetorical analysis of social representations of COVID‐19 allowed us to examine their forms (visual strategies) as well as their functions. This kind of analysis answers to the call to focus more on action‐orientation instead of object‐orientation to emphasise the functionality of social representations in shaping social and political relations (Batel & Castro, 2018: Buhagiar & Sammut, 2020). Our analysis of visual rhetoric shows how visual strategies were used in these images to construct a particular stance regarding different age groups and create subject positions for these groupings with the intention of directing perception and contributing to meaning. When similar kinds of images are repeated in media, they shape and may eventually naturalise our understanding of and relation to different age groups. Hence, newspaper images may not only disseminate but also naturalise social representations of different age groups (Moscovici, 1961/2008, 1984). The usefulness of the analysis of visual rhetoric in social representations research is based on its capability to unfold the constructed nature of images and the social representations they establish and maintain (Martikainen, 2019). This is especially important for news photography in particular, since these images are often regarded as replicas of reality and objective evidence of news (Banks, 2012; Zelizer, 1995). Because of the institutional status of media, media images significantly contribute to interpersonal and intergroup relations, solidarity vs. antagonism and social stability vs. community conflict. Therefore, it is important to promote the development of skills of (critical) visual literacy in all age groups and walks of life.

In this study, our focus was on the subject positions that were constructed for different age groups in relation to social representations of COVID‐19. In line with the basic idea of subject position, which holds that every position has a moral quality and is imbued with a set of rights and duties, we were able to investigate the ways in which power was intertwined with the representational process. The findings showed that children were depicted as controlled pupils and carefree players, which positioned them as bystanders with less direct connection to COVID‐19 than the other age groups. Youth, in turn, were depicted as future‐oriented graduates and reckless partygoers. The latter depiction positioned them as a potential risk to the health of the community. Adults were depicted as authoritative experts, adaptive professionals, responsible caretakers and active recreationists, positioning them as bearing the responsibility of keeping society running. Finally, elderly people were depicted as separated from their loved ones and social activities, which positioned them as isolated loners.

In the future, it would be interesting to study how different age groups are represented in media images in other countries, not only in newspaper images but also in diverse forms of social media.

Hence, echoing Páez and Pérez's (2020) study on COVID‐19 and Wagner‐Egger et al.'s (2011) study on H1N1, which identified positions of heroes/saviours, villains and victims during these outbreaks, our findings suggest that youth (in the subject position of reckless partygoers) were positioned as carefree villains, adults as heroes and elderly people as victims. In addition, the large difference in the number of photographs depicting adults, the more diverse subject positions constructed for them, the overall position of adults being in charge of society's functioning, and the relatively few images depicting different age groups together seem to construct a divide between adults and the other age groups. Simultaneously, the images positioned adults as having power, whereas children, youth and the elderly were depicted by a lack of power. In line with Joffe (2011), who argued that collective coping with emerging diseases occurs through a three‐phased pattern, it remains to be seen if reckless young people will continue to be stigmatised and treated as responsible for the spread of the virus.

## CONFLICT OF INTEREST

The authors declare there is no conflict of interest.

## ETHICAL STATEMENT

The manuscript adheres to the editorial policies and ethical considerations of specified by the *Journal of Community and Applied Social Psychology*. The research is conducted ethically, results are reported honestly, the submitted work is original and not (self‐)plagiarised and authorship reflects individuals' contributions. The study adheres to ethical guidelines of the National Advisory Board on Research Ethics (TENK) in Finland and Declaration of Helsinki. No ethics approval statement is required for this study. Permission to reproduce material from other sources: In our article, we cite images published in *Helsingin Sanomat* and *Ilta‐Sanomat* newspapers. We refer to the guidelines of Finnish copyright law (image citation) according to which images of published photographs can be included in a scientific presentation when the following criteria are met: images must be related to the text; they must illustrate and clarify the text; the creator's name, the year and the location of the image (newspaper and details of the issue) are given.

## Data Availability

Data derived from public domain resources https://www.hs.fi/lehdet/ and https://www.is.fi/lehti/
